# *TCF7L2* lncRNA: a link between bipolar disorder and body mass index through glucocorticoid signaling

**DOI:** 10.1038/s41380-021-01274-z

**Published:** 2021-09-17

**Authors:** Duan Liu, Thanh Thanh Le Nguyen, Huanyao Gao, Huaizhi Huang, Daniel C. Kim, Brenna Sharp, Zhenqing Ye, Jeong-Heon Lee, Brandon J. Coombes, Tamas Ordog, Liewei Wang, Joanna M. Biernacka, Mark A. Frye, Richard M. Weinshilboum

**Affiliations:** 1 Department of Molecular Pharmacology and Experimental Therapeutics, Mayo Clinic, Rochester, MN USA; 2 Graduate School of Biomedical Sciences, Mayo Clinic, Rochester, MN USA; 3 Division of Computational Biology, Department of Quantitative Health Sciences, Mayo Clinic, Rochester, MN USA; 4 Department of Laboratory Medicine and Pathology, Mayo Clinic, Rochester, MN USA; 5 Department of Physiology and Biomedical Engineering, Mayo Clinic, Rochester, MN USA; 6 Division of Gastroenterology and Hepatology, Department of Internal Medicine, Mayo Clinic, Rochester, MN USA; 7 Department of Psychiatry and Psychology, Mayo Clinic, Rochester, MN USA

**Keywords:** Genetics, Molecular biology

## Abstract

Bipolar disorder (BD) and obesity are highly comorbid. We previously performed a genome-wide association study (GWAS) for BD risk accounting for the effect of body mass index (BMI), which identified a genome-wide significant single-nucleotide polymorphism (SNP) in the gene encoding the transcription factor 7 like 2 (*TCF7L2*). However, the molecular function of *TCF7L2* in the central nervous system (CNS) and its possible role in the BD and BMI interaction remained unclear. In the present study, we demonstrated by studying human induced pluripotent stem cell (hiPSC)-derived astrocytes, cells that highly express *TCF7L2* in the CNS, that the BD-BMI GWAS risk SNP is associated with glucocorticoid-dependent repression of the expression of a previously uncharacterized TCF7L2 transcript variant. That transcript is a long non-coding RNA (lncRNA-TCF7L2) that is highly expressed in the CNS but not in peripheral tissues such as the liver and pancreas that are involved in metabolism. In astrocytes, knockdown of the lncRNA-TCF7L2 resulted in decreased expression of the parent gene, *TCF7L2*, as well as alterations in the expression of a series of genes involved in insulin signaling and diabetes. We also studied the function of TCF7L2 in hiPSC-derived astrocytes by integrating RNA sequencing data after TCF7L2 knockdown with TCF7L2 chromatin-immunoprecipitation sequencing (ChIP-seq) data. Those studies showed that TCF7L2 directly regulated a series of BD risk genes. In summary, these results support the existence of a CNS-based mechanism underlying BD-BMI genetic risk, a mechanism based on a glucocorticoid-dependent expression quantitative trait locus that regulates the expression of a novel TCF7L2 non-coding transcript.

## Introduction

Bipolar disorder (BD) is a psychiatric disease with significant morbidity and mortality [1]. BD is often comorbid with other disorders such as alcohol use disorder [2–4] and binge eating disorder [5]. The overall heritability of BD is between 60% and 85%, indicating that genetic factors contribute substantially to disease risk [6]. Genome-wide association studies (GWAS) have identified >60 genome-wide significant loci associated with BD risk [6–13]. Pathway enrichment analyses of BD risk genes have revealed several significant gene sets, including those that regulate insulin secretion, energy metabolism [6, 7], and corticotropin-releasing hormone (CRH) signaling [14]. Those results are consistent with clinical observations that BD is often associated with binge eating disorder [5], addiction disorders [2–4, 15] (i.e., alcohol and food), and with metabolic syndromes [16]. The prevalence of obesity in BD patients is more than twofold greater than that for the general population [16, 17]. Furthermore, obesity in BD is associated with more severe mood symptoms and worse treatment outcomes [18, 19]. The pathophysiology underlying the comorbidity of BD and obesity remains unclear.

In an attempt to gain a greater understanding of the potential contribution of genetic factors to the comorbidity of BD and obesity, we performed a GWAS to identify variants associated with body mass index (BMI)-dependent BD [20]. Even though BMI is an imperfect measure of excessive fat accumulation, it is a widely used clinical variable in studies of obesity [21, 22]. That GWAS identified a genome-wide significant single-nucleotide polymorphism (SNP) (rs12772424, *P* = 2.85E-08) that mapped to an intronic region of the *TCF7L2* gene [20]. Specifically, the minor allele for the rs12772424 SNP was more strongly associated with BD as BMI increased [20]. However, this SNP was not associated with risk for either BD or BMI alone, but only showed a significant association with BD when accounting for the effect of BMI [20]. The association of this *TCF7L2* SNP with BMI-dependent BD risk was later replicated in an independent cohort of BD patients [23]. *TCF7L2* is a well-known risk gene for type 2 diabetes (T2D). Specifically, a different *TCF7L2* SNP (rs7903146)—a SNP that is not in linkage disequilibrium (LD) with rs12772424 (*r*^*2*^ < 0.035, *D*’ <0.282) and which maps 122.2 kb distant from rs1277424—has been associated with risk for T2D in a series of studies [24–27]. The existence of two independent *TCF7L2* SNPs, each of which is associated with different diseases, suggests that *TCF7L2* might have different functions in T2D and in BD associated with BMI.

*TCF7L2* encodes the transcription factor 7 like 2, which plays an important role in the Wnt/β-catenin signaling pathway [28], a pathway known to be involved in the pharmacotherapy of BD [29]. *TCF7L2* is widely expressed across many human organs and tissues, including the brain [30]. As *TCF7L2* is well recognized as a T2D risk gene, its function in T2D and glucose metabolism has been studied extensively in animal models with results that suggest a complex molecular role for this protein [31]. Specifically, *Tcf7l2* dysfunction in hepatic [32–34] and pancreatic [35, 36] tissue, as well as involvement in the gut-brain axis [37] have all been thought to contribute to T2D risk. *Tcf7l2* function in the central nervous system (CNS) has also been studied in rodents [38–40]. In addition to disrupted systemic glucose homeostasis, abnormalities in nervous system morphology and neurologically based behaviors such as polyphagia and hypoactivity were observed in *Tcf7l2* knockout mice [41, 42]. Despite mounting evidence that *TCF7L2* may link psychiatric disorders and metabolic dysregulation [20, 43], its function in human CNS cell line models, to our knowledge, has not been reported.

In the present study, we set out to characterize the molecular function of *TCF7L2* in the CNS with the goal of understanding its possible role in the comorbidity of BD and obesity. These studies began with a review of human brain single-nucleus and single-cell RNA-seq data for TCF7L2, results which showed that it is most highly expressed in astrocytes. Furthermore, an examination of the genomic architecture surrounding the BD-BMI interaction GWAS identified SNP, rs12772424, showed that the variant SNP genotype created a half-palindrome glucocorticoid response element (GRE)—raising the possibility that *TCF7L2* transcription might be regulated by glucocorticoid signaling. After treatment of hiPSC-derived astrocytes with dexamethasone (DEX), a potent synthetic glucocorticoid, expression levels of two TCF7L2 long non-coding transcripts (lncRNA-TCF7L2) were found to be significantly repressed. One of the lncRNA-TCF7L2 transcripts, namely “T-3”, was predominantly expressed in the human brain but not in peripheral tissues such as the liver or pancreas. As a result, after starting with an SNP that was associated with a BD subtype, the series of experiments described subsequently showed that the SNP was a pharmacogenomic expression quantitative trait locus (PGx-eQTL), i.e., an eQTL that is functional only in the presence of a drug (e.g., DEX) or endogenous hormones such as glucocorticoids [44–46]. Finally, we found that the “T-3” lncRNA-TCF7L2 influenced expression in astrocytes of both the parent gene and scores of other genes involved in both energy metabolism and BD risk. This series of studies identified and functionally characterized a TCF7L2 lncRNA and its SNP-dependent expression as a molecular mechanism related to BD-BMI interaction and, as a result, illustrates the potential importance of lncRNAs for neuropsychiatry.

## Materials and methods

### TCF7L2 transcriptional analysis in the human brain at the single-cell level

A single-nucleus RNA sequencing (snRNA-seq) data set generated by the Allen Institute that included samples across multiple human cortical areas [47] was consulted to obtain information with regard to TCF7L2 expression in CNS cells. In addition, single-cell RNA sequencing (scRNA-seq) data generated using surgically removed human cerebral cortical samples from 12 subjects [48, 49] were downloaded and combined for analysis of TCF7L2 expression. Finally, chromatin accessibility information for the *TCF7L2* gene in human brain cells was obtained from a single-cell assay for transposase-accessible chromatin using sequencing (scATAC-seq) data set [50] to evaluate the transcriptional activity of *TCF7L2* in clustered CNS cell types. The sources of these data sets and other key resources used in this study are listed in Supplementary Table S1. Please see the Supplementary Text for details.

### Identification of the TCF7L2 transcript variants regulated by glucocorticoid signaling

The *TCF7L2* rs12772424 SNP was annotated using HaploReg v4.1 [51], which indicated that the rs12772424 variant allele (A) created a half-palindrome GRE. To test the hypothesis that TCF7L2 transcription might be regulated by glucocorticoid signaling, quantitative reverse transcription-polymerase chain reaction (qRT-PCR) was used to quantitate RNA levels for all known TCF7L2 transcript variants in a variety of cell lines before and after treatment with the glucocorticoid receptor (GR) agonist DEX. The rs12772424 SNP effect on *TCF7L2* transcriptional activity was also determined by reporter gene assay with the comparison of luciferase activities for rs12772424 SNP wild-type and variant cDNA constructs. See the Supplementary Text for details.

### Characterization of TCF7L2 long non-coding RNA transcripts

Expression levels of TCF7L2 long non-coding RNA transcripts (lncRNA-TCF7L2) in the human brain and peripheral tissues as well as in human cell lines were quantified by qRT-PCR using primers targeted to their unique exon junctions. The existence of lncRNA-TCF7L2 “T-3” was confirmed by cDNA amplification of its “full length” sequence using total RNA samples prepared from human brain tissues and from hiPSC-derived astrocytes. RNA-seq was also performed with samples from hiPSC-derived astrocytes that have been transfected with ASOs to knockdown (KD) the lncRNA-TCF7L2 or with non-targeting control ASOs. Differentially expressed genes (DEGs) after KD of the lncRNA-TCF7L2 “T-3” were identified by comparison of transcriptomes of control and KD samples. Those DEGs were then used for pathway enrichment analysis using EnrichR [52] to annotate the function of the lncRNA-TCF7L2 “T-3”. See the *Supplementary Text* for additional details.

### Identification of TCF7L2 target genes in hiPSC-derived astrocytes

RNA-seq and ChIP-seq data were generated to identify TCF7L2 target genes. Specifically, TCF7L2 protein expression was knocked down (KD) by siRNA transfection. After the KD efficiency had been validated by both qRT-PCR and western blot assays, RNA-seq was performed with control and KD samples to identify transcriptome-wide DEGs. ChIP-seq was also performed to identify TCF7L2 genome-wide DNA-binding sites. RNA-seq and ChIP-seq data were integrated as described previously [53] for the identification of TCF7L2 target genes. See the Supplementary Text for details.

### TCF7L2 and BD risk genes

To annotate TCF7L2 protein function, the RNA-seq and ChIP-seq identified TCF7L2 target genes in hiPSC-derived astrocytes were subjected to pathway enrichment analysis. Those TCF7L2 target genes enriched BD as the top disease pathway. Expression levels of the BD risk genes quantified by RNA-seq were plotted, and the TCF7L2-binding sites that mapped to or near the BD risk genes identified by ChIP-seq were visualized by using the Integrative Genomics Viewer [54]. See the Supplementary Text for details.

## Results

### *TCF7L2* gene transcription in human brain cells

**Fig. 1:**
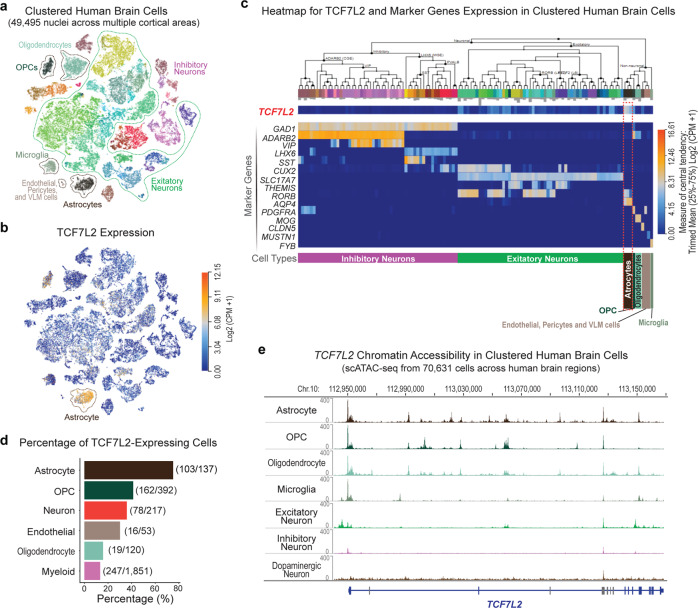
*TCF7L2* transcription in the human brain. **a** Clusters of human brain cells that were assigned cell types based on the expression of cell type-specific marker genes quantified by single-nucleus RNA-seq in 49,495 single nuclei from multiple cortical areas. Each dot represents a single cell. Cell types have been color-coded and labeled. OPCs: oligodendrocyte progenitor cells. **b** TCF7L2 expression in each of these brain cells was color-coded based on TCF7L2 expression levels. **c** Heatmap for the bulk expression level of *TCF7L2* and marker genes in the clustered brain cell. Expression level in astrocytes is highlighted in the dashed box. **a**–**c** show data generated by the Allen Institute (https://portal.brain-map.org/atlases-and-data/rnaseq) [47]. **d** Percentages of TCF7L2-expressing cells in each sub-group of cell types are shown graphically. Numbers shown in parentheses are the numbers of TCF7L2-expressing cells/total number of sequenced cells. Single-cell RNA-seq data were generated from surgically removed human cerebral cortical tissues in two studies [48, 49]. The two data sets were combined and re-analyzed as reported in a previous study [55]. **e** *TCF7L2* chromatin accessibility was quantified by single-cell ATAC-seq in 70,631 cells across human brain regions. ATAC-seq peaks across the *TCF7L2* gene in each clustered cell type are shown separately from top to bottom. Physical positions on chromosome 10 (Chr.10) are labeled across the top. Based on its physical location on Chr.10 (GRCh38/p13), the *TCF7L2* gene is depicted at the bottom with exons shown as bars and introns as lines. The scATAC-seq data were generated by Corces et al. [50] and visualized by using the WashU Epigenome Browser [81].

To determine which CNS cell type(s) might express TCF7L2, we first consulted human brain snRNA-seq data generated by the Allen Institute [47]. Their data set included single-nucleus transcriptomes of 49,495 nuclei across multiple human brain cortical areas. Based on marker gene expression, cells were “clustered” into seven non-neuronal and two major neuronal cell types (Fig. 1a). TCF7L2 was more highly expressed in astrocytes than in any of the other “clustered” cell types (Fig. 1b, c). Previously we had also analyzed scRNA-seq data [55] from two data sets generated from surgically removed human cerebral cortical tissue [48, 49] (Supplementary Fig. S1). In those studies, TCF7L2 was also expressed predominantly in astrocytes, e.g., 75.2% of astrocytes were TCF7L2 positive, a percentage nearly twice that of any other CNS cell type (Fig. 1d). The high levels of TCF7L2 expression in astrocytes were supported by scATAC-seq data generated from 70,631 human brain cells [50]. The human brain scATAC-seq data showed that *TCF7L2* chromatin accessibility in astrocytes was higher than that for other clustered CNS cell types (Fig. 1e), compatible with highly active *TCF7L2* transcription in astrocytes. Since *TCF7L2* is most highly expressed in astrocytes, and since insulin signaling in astrocytes is known to co-regulate CNS glucose sensing and systemic glucose metabolism [56, 57], we next set out to characterize *TCF7L2* function in hiPSC-derived astrocytes in an effort to understand its possible role in the comorbidity of BD and obesity.

### The BMI-dependent BD risk SNP regulates brain-specific *TCF7L2* transcript variant expression through glucocorticoid signaling

**Fig. 2:**
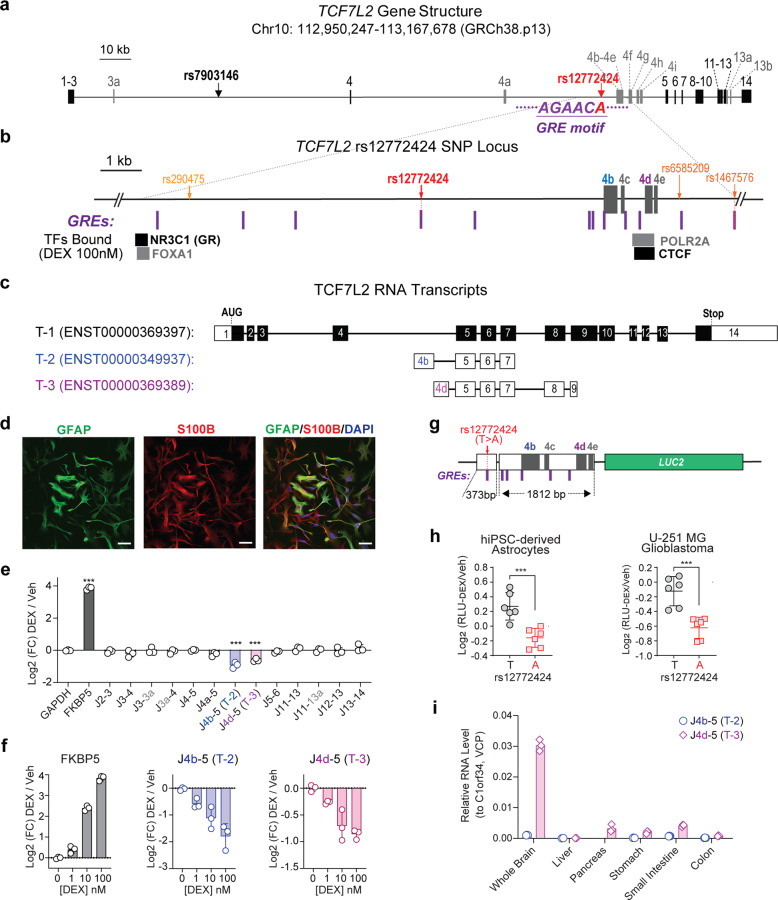
*TCF7L2* SNPs and glucocorticoid-regulated transcript variants. **a** *TCF7L2* gene structure. *TCF7L2* exons are numbered from 1 to 14 based on the reference RNA transcript (ID: ENST00000369397). Alternative exons that map to introns of the reference transcript were named using the intron number, followed by a letter of the alphabet. Positions of the rs7903146 SNP and the rs12772424 SNP are indicated and they map 122.2 kb distant from each other. The rs12772424 SNP variant allele (*A*) created a half-palindrome glucocorticoid response element (GRE). **b** The rs12772424 SNP locus. Positions of rs12772424 and its “linked” SNPs (*r*^*2*^ > 0.35, *D*’ >0.79 in European population) are labeled. *TCF7L2* exons are depicted as boxes with exon numbers labeled above. Putative GREs are indicated below. Transcription factor (TF) binding sites for the glucocorticoid receptor (NR3C1), FOXA1, POLR2A, and CTCF are shown as boxes below the GREs. Darker colors represent stronger binding. **c** TCF7L2 transcript variants (T-1 to T-3) annotated by *Ensembl* are shown with their IDs shown in parentheses. **d** Immunofluorescent staining of astrocyte markers, GFAP and S100B, for hiPSC-derived astrocytes. Scale bars represent 50 µM. **e** Fold changes in RNA levels for *TCF7L2* transcript variants in hiPSC-derived astrocytes after treatment with 100 nM DEX. Unique exon junctions (“J”) for specific transcript variants were quantified by qRT-PCR. *GAPDH* and *FKBP5* expression was measured as internal and treatment controls, respectively. Data are mean ± s.d. (*n* = 3). ***P < 0.001 by Dunnett’s test. **f** DEX-dose-dependent induction of FKBP5 and repression of “T-2” and “T-3” in hiPSC-derived astrocytes. **g** Construction of reporter gene plasmid. DNA fragments that included the *TCF7L2* rs12772424 SNP (373 bp) and exons 4b-4d (1812 bp) were cloned into the 5′-region of the *LUC2* reporter gene. **h** Luciferase assays after cells were transfected with plasmids containing rs12772424 wild type (*T*) or variant (*A*) alleles and were treated with DEX or vehicle control. Data are presented as relative luciferase units (RLUs) after dexamethasone/vehicle (DEX/Veh) normalization. ***P < 0.001 by *t* test. **i** Relative RNA levels of TCF7L2 “T-2” and “T-3” compared with those of the housekeeping genes, *VCP* and *C1orf43*, in total RNA samples prepared from human tissue lysates. Data are mean ± s.d. (*n* = 3).

We began this series of experiments by attempting to understand the *TCF7L2* gene structure and the SNP signal that had initially suggested a possible relationship of *TCF7L2* to BD risk. The *Ensembl* [58] human genome assembly (GRCh38/p13) annotated 26 human *TCF7L2* exons (numbered in Fig. 2a) which gave rise to 30 possible transcript variants (see Supplementary Tables S2 and S3). The most highly expressed TCF7L2 transcript across human tissues is a protein-coding transcript (ENST00000369397) consisting of 14 exons [30]. We designated that transcript as the TCF7L2 reference transcript (Fig. 2a). Alternative exons that mapped to intronic regions of the reference transcript were named on the basis of intron number, followed by a letter of the alphabet (see Fig. 2a). The *TCF7L2* rs12772424 SNP that was associated with the BD-BMI interaction is independent of the *TCF7L2* rs7903146 T2D risk SNP, e.g., they are not in LD in any racial or ethnic groups (*r*^*2*^ < 0.035, *D*’ <0.282). Unlike the T2D risk rs7903146 SNP, which is an eQTL for TCF7L2 RNA levels in multiple tissues [30], the rs12772424 SNP is not an eQTL for TCF7L2 in any tissue or organ [30]. However, we noticed that the variant allele for the rs12772424 SNP created a half-palindrome GRE (see Fig. 2a), a DNA motif that binds to the ligand-activated GR, which could then either enhance or repress gene transcription [59]. We also noted that eleven additional GREs were present in the “LD block” that includes rs12772424 (Fig. 2b). To determine whether the GR or other GR-related transcription factors (TFs) might bind to this “LD block”, we consulted the ENCODE [60] database that contains ChIP-seq data generated using A549 lung cancer cells after treatment with the GR agonist, DEX. Those ChIP-seq data showed that GR (encoded by *NR3C1*), FOXA1, POLR2A, and CTCF were all bound to this haplotype block (Fig. 2b). These observations raised the possibility that *TCF7L2* transcription might be influenced by glucocorticoid signaling.

To determine whether glucocorticoids might regulate *TCF7L2* transcription, we designed qRT-PCR primers that targeted all known *TCF7L2* exon junctions (J) (see Supplementary Table S4) to make it possible to quantitate all TCF7L2 RNA transcripts after DEX treatment. The reference TCF7L2 transcript variant is designated “T-1” in Fig. 2c. More than half of the 30 *Ensembl*-annotated TCF7L2 transcript variants failed to include exons 1–4 of “T-1”. Furthermore, as we will demonstrate subsequently, the expression of two “short” variant transcripts, namely “T-2” and “T-3” (see Fig. 2c), were significantly repressed by DEX treatment.

As a first step, we measured RNA levels for all annotated *TCF7L2* transcripts in A549 cells since ChIP-seq showed that GR and other TFs bound near the rs12772424 SNP in A549 cells after treatment with 100 nM DEX (Fig. 2b). The RNA level of *FKBP5*, a prototypic GR-inducible gene, was also assayed as a positive control for response to DEX treatment. As expected, FKBP5 RNA expression increased significantly after DEX treatment (Supplementary Fig. S2a). However, the expression of three of the possible TCF7L2 transcript variants, including “T-2” and “T-3”, was significantly downregulated, whereas the expression of other TCF7L2 potential transcript variants was not changed significantly (Supplementary Fig. S2a) nor were they detectable by qRT-PCR (data not shown). Furthermore, repression of the “T-2” and “T-3” transcripts was DEX-dose dependent (Supplementary Fig. S2b). In an attempt to validate these observations for A549 cells by using CNS cell lines, we cultured human iPSC-derived astrocytes (Fig. 2d) and once again showed that “T-2” and “T-3” expression was repressed by DEX treatment (Fig. 2e) in a dose-dependent fashion (Fig. 2f). These observations were also reproduced in U251-MG human glioblastoma cells (Supplementary Fig. S2c, d), indicating that the regulation of those TCF7L2 transcript variants by glucocorticoid signaling may be a general phenotype across cell lines.

To study the possible role of the rs12772424 SNP in GR-dependent repression of “T-2” and “T-3” in the presence of DEX, DNA fragments containing the rs12772424 SNP and exon 4b-4d sequences were cloned into luciferase reporter gene constructs (see Fig. 2g). Constructs containing the rs12772424 SNP variant allele (A), which creates a GRE motif, were associated with decreased luciferase activity in both iPSC-derived astrocytes and U251-MG glioblastoma cells after DEX treatment (Fig. 2h), suggesting that the transcriptional activity of the cloned DNA fragments was GR and rs12772424 SNP-dependent in both types of cells.

Finally, “T-3” RNA was much more abundant in total RNA samples obtained from the human brain than in total RNA isolated from liver, pancreas, or gut tissue (Fig. 2i), tissues in which the function of *TCF7L2* may be linked to T2D [32–37]. Compared with “T-3”, RNA levels of “T-2” were very low or undetectable in all tissues that were tested (Fig. 2i). In summary, this series of experiments suggested that the *TCF7L2* transcript variant “T-3”, which is highly expressed in the human brain but not in the other tissues that we tested, might be linked to the BD-BMI risk SNP rs12772424 through GR signaling—a possibility supported by the fact that the rs12772424 SNP creates a GRE.

### TCF7L2 “T-3” is a long non-coding RNA transcript that functions in the regulation of TCF7L2 mRNA and of genes involved in metabolic pathways

**Fig. 3:**
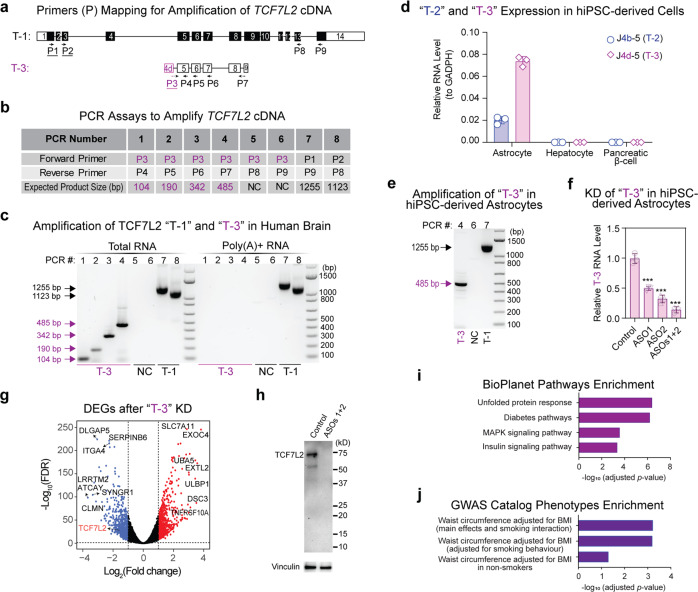
Characterization of TCF7L2 Transcript “T-3”. **a** *TCF7L2* transcripts “T-1” and “T-3” and mapping sites of primers (P) that were used to amplify *TCF7L2* cDNA. Primer mapping sites are labeled by arrows below the exons depicted as boxes in which exon numbers are labeled. Primers (P) are numbered from 5′ to 3′ of the gene. Forward primers are underlined. Primer sequences are listed in Supplementary Table S5. **b** The table lists eight polymerase chain reactions (PCR) used to amplify portions of the *TCF7L2* cDNA. The combination of primers for each PCR reaction and the estimated base pair (bp) length of the PCR product are listed. NC = negative control reaction, which was not expected to yield a PCR product. **c** RT-PCR products using human brain total RNA or poly(A) + RNA as templates are shown after separation on a 1.2% agarose gel. PCR numbers (#) match the PCR assays listed in **b**. PCR assays that were designed to amplify “T-3” (from #1 to #4) yielded products of the expected sizes when total RNA was used as the PCR template but failed to amplify target sequences when poly(A) + RNA was the template. NC reactions (#5 and #6) and positive control reactions (#7 and #8) that amplify *TCF7L2* mRNA transcript “T-1” produced the expected results. **d** Relative RNA levels of TCF7L2 “T-2” and “T-3” compared with GAPDH in hiPSC-derived astrocytes, hepatocytes, and pancreatic β-cells. Data are mean ± s.d. **e** RT-PCR products amplified TCF7L2 cDNA in hiPSC-derived astrocytes. **f** qRT-PCR determined “T-3” levels in hiPSC-derived astrocytes after transfection with antisense oligonucleotides (ASOs) targeting exon-4d. The *Y* axis represents “T-3” expression relative to control (non-targeting ASO). The combination of ASO 1 and 2 (ASOs 1+2) resulted in the best knockdown (KD) efficiency. **g** Volcano plot for RNA-seq identified differentially expressed genes (DEGs) in hiPSC-derived astrocytes. TCF7L2 mRNA expression was downregulated after “T-3” KD. **h** Western blot assays for TCF7L2 after knockdown of lncRNA-TCF7L2 “T-3” in hiPSC-derived astrocytes. **i** Top pathways enriched by DEGs of “T-3” KD (FC > 2.0; FDR < 0.05) in BioPlanet data set and **j** GWAS Catalog phenotypes.

Transcript variant “T-3” was distinguished from other *TCF7L2* transcripts by its first exon, exon-4d (Fig. 3a). In addition to quantification of the “T-3” unique exon junction, “J4d-5”, by the use of qRT-PCR (Fig. 2), we were also able to amplify all six “T-3” exons by RT-PCR using primers mapping to “T-3” (see Supplementary Table S5) and total RNA prepared from human brain tissue as a template (Fig. 3a–c). This result confirmed the existence of “T-3” in the human brain. We should note that “T-3” has been annotated as a protein-coding transcript without a stop codon or a 3′ untranslated region (UTR) but—based on our results—it appears to be a *TCF7L2* long non-coding RNA transcript (lncRNA-TCF7L2) in the human brain. That conclusion is supported by the western blot assays shown subsequently, which failed to detect the putative protein band (~18 kD) that would be encoded by “T-3” and by the fact that PCR performed with primers targeting “T-3” failed to amplify a product when cDNA synthesized from human brain poly(A) + RNA was used as template (Fig. 3c). This result also explains why, when RNA-seq is performed with poly(A)+ selected cDNA libraries which capture mainly protein-coding RNA, including the libraries used by GTEx [30] and the scRNA-seq data that we analyzed [48, 49], “T-3” could not be detected.

In an effort to study the possible function of “T-3”, we first quantified “T-3” expression in hiPSC-derived astrocytes, hepatocytes, and pancreatic β cells (cell characterization shown in Supplementary Fig. S3), which showed that “T-3” was highly expressed in hiPSC-derived astrocytes but not in two peripheral cell models (Fig. 3d). The presence of “T-3” in hiPSC-derived astrocytes was further confirmed by cDNA amplification using total RNA as a template (Fig. 3e). “T-3” was then KD in hiPSC-derived astrocytes by using antisense oligonucleotides (ASO) targeting exon-4d. The “T-3” KD efficiency was >80% in astrocytes transfected with pooled ASOs (Fig. 3f). That RNA sample, together with a non-targeting ASO control, was then used to perform RNA-seq. “T-3” KD in astrocytes resulted in a total of 1363 DEGs with fold change (FC) ≥ 2 (FDR < 0.05), which included *TCF7L2* poly(A)+/mRNA (see Fig. 3g and Supplementary Table S6). Western blot analysis further confirmed the decrease in TCF7L2 protein after “T-3” KD in hiPSC-derived astrocytes (Fig. 3h), an observation that was reproduced in two additional cell lines that express “T-3” (Supplementary Fig. S4). When the DEGs after T-3 KD were used to perform pathway enrichment analysis using Enrichr [52], the “top” enriched pathway in the BioPlanet database [61] was “unfolded protein response” (Fig. 3i), an endoplasmic reticulum stress response pathway which is well-known to play a role in diabetes, metabolic syndrome and neurodegenerative disorders [62]. Diabetes and insulin signaling pathways were also identified by the pathway enrichment analysis (Fig. 3i). Those DEGs also identified “waist circumference adjusted for BMI” as the “top” associated phenotype in the GWAS Catalog [63] (Fig. 3j), a phenotype that is a clinical measure related to obesity and related disease risk [64]. In summary, we experimentally validated the existence of the TCF7L2 long non-coding transcript “T-3” in the human brain and, particularly, in hiPSC-derived astrocytes. The RNA-seq analysis after “T-3” KD indicated that this lncRNA might contribute to the regulation of the expression of genes related to metabolic phenotypes, a link to the BD-BMI phenotype identified during our original GWAS in which rs12772424 was the “top hit” SNP.

### Integration of TCF7L2 RNA-seq and ChIP-seq identified genes that play a role in CNS development

**Fig. 4:**
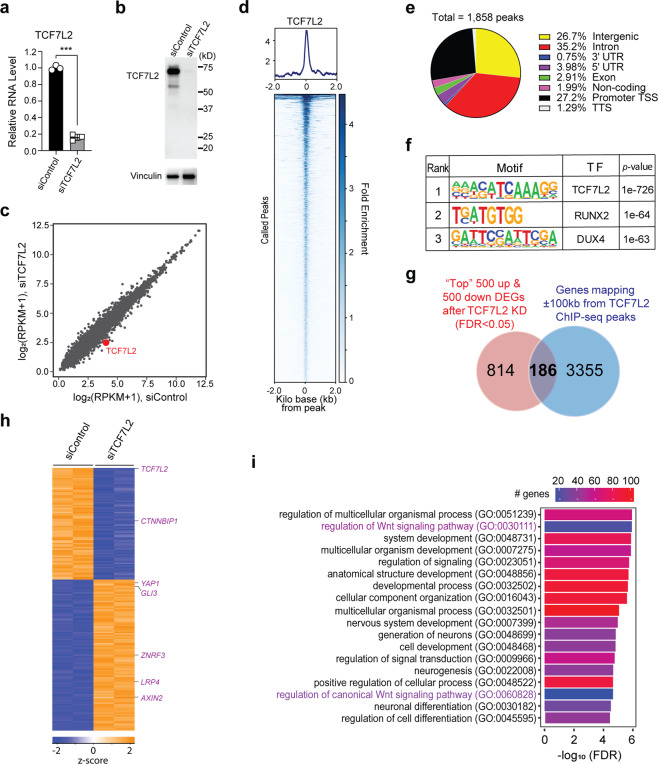
TCF7L2 protein function in hiPSC-derived astrocytes. **a** TCF7L2 RNA levels in hiPSC-derived astrocytes after transfection with non-targeting siRNA control (siControl) or “pooled” TCF7L2-targeting siRNAs (siTCF7L2). **b** Western blot assay for TCF7L2. Vinculin was blotted as an internal control. **c** Expression levels as log_2_ (reads per kilobase of transcript per million reads mapped [RPKM] +1) values for all detected genes in the RNA-seq libraries for siTCF7L2 (*Y* axis) compared with siControl (*X* axis). *n* = 2 independent replicates for both groups. **d** Heatmap of TCF7L2 ChIP-seq results, in which regions covering the called peaks ±2 kb were scaled to the same size relative to the center point of the peaks. The heatmap intensity represents fold enrichment of the TCF7L2 ChIP signal over input, with each row depicting each called peak, and with the *X* axis representing position relative to the center point of the peaks. The top panel is the average profile of the ChIP-seq peaks, with the *Y* axis representing fold enrichment of the TCF7L2 ChIP signal over input. **e** Distribution of 1858 identified TCF7L2 peaks (FDR < 0.01) across the genome. *UTR* untranslated region, *TSS* transcription start site, *TTS* transcription termination site. **f** The top three binding motifs and their binding transcription factors (TFs) were identified from HOMER de novo analysis based on *p* values. **g** Integration of RNA-seq and ChIP-seq data identified 186 genes that were both bound by TCF7L2 and were differentially expressed after TCF7L2 KD. Based on false discovery rate (FDR < 0.05), the “top” 500 up- and 500 downregulated DEGs and genes mapping ±100 kb from the TCF7L2 ChIP peaks (3541 genes) were used to identify “overlapping” TCF7L2 target genes. **h** Heatmap of expression for the 186 TCF7L2 target genes in control and TCF7L2 KD samples. Genes involved in the Wnt signaling pathways have been labeled. **i** Gene Ontology enrichment analysis for the 186 TCF7L2 target genes identified by integration of the RNA-seq and ChIP-seq data. Colors represent the number of genes enriched in each pathway. The *X* axis represents −log_10_ of the FDR. Wnt signaling pathways have been highlighted.

TCF7L2 mRNA was more highly expressed in astrocytes than in other CNS cell types (Fig. 1). However, the function of TCF7L2 as a TF in astrocytes remained unclear. Since TCF7L2 mRNA and protein levels were significantly decreased after “T-3” KD (Fig. 3g, h), we next performed TCF7L2 RNA-seq and ChIP-seq to identify TCF7L2 target genes in hiPSC-derived astrocytes. TCF7L2 mRNA was efficiently (>80%) KD by siRNA (Fig. 4a), which mainly targets cytoplasmic mRNA [65]. KD of TCF7L2 protein was confirmed by western blot assay (Fig. 4b). DEGs were identified by the comparison of RNA-seq data obtained from astrocytes transfected with non-targeting control and those transfected with TCF7L2 siRNAs (Fig. 4c). We also performed TCF7L2 ChIP-seq to identify the DNA-binding sites for this TF across the genome of hiPSC-derived astrocytes (Fig. 4d). A total of 1858 TCF7L2 ChIP peaks were identified, approximately one-third of which mapped to promoters and transcription start and termination sites (TSS and TTS) (Fig. 4e). As expected, DNA motif analysis identified the prototypic TCF7L2-binding motif as the “top hit”, supporting the specificity of the ChIP-seq results (Fig. 4f). The ChIP-seq peaks were then integrated with RNA-seq-identified DEGs [53] to identify genes directly targeted by TCF7L2 (Fig. 4g). A total of 186 “top” DEGs after TCF7L2 KD that bound TCF7L2 were identified based on the ChIP-seq data (Fig. 4g, Supplementary Table S7). Those 186 genes included *TCF7L2* itself, which was previously known to be bound by TCF7L2 protein [66], the *AXIN2* gene that is a prototypic Wnt target gene [67], and other genes that are known to be involved in Wnt signaling (Fig. 4h). When the 186 TCF7L2 target genes in hiPSC-derived astrocytes were subjected to pathway enrichment analysis, as expected, the Wnt signaling pathway was identified as one of the “top” biological pathways (Fig. 4i). In addition, developmental pathways, including nervous system development and neurogenesis pathways, were enriched by the 186 TCF7L2 target genes (Fig. 4i), a result consistent with the known role of TCF7L2 in Wnt signaling and in cell development [28]. These results provided evidence which suggested that TCF7L2 may play a role in human CNS development, a process that has been linked to BD pathophysiology [68].

### TCF7L2 directly regulates BD risk genes

**Fig. 5:**
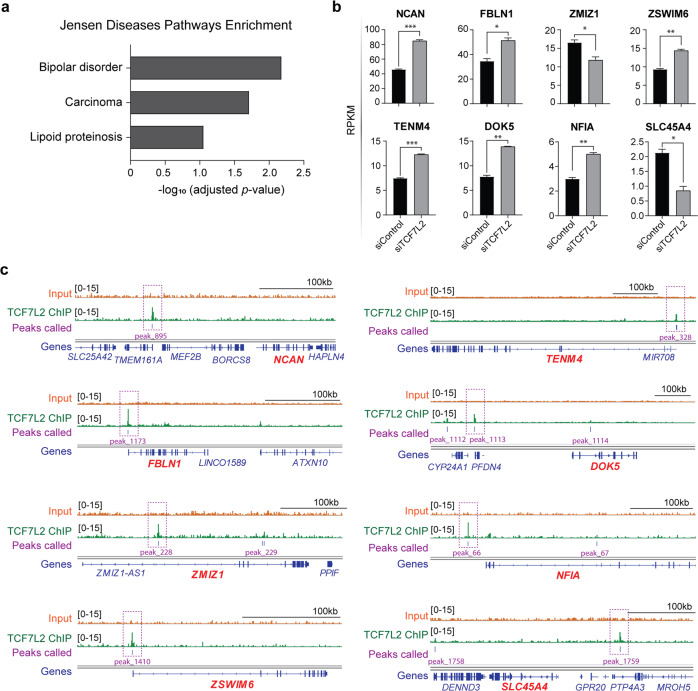
TCF7L2 directly regulates BD risk genes. **a** Disease pathway enrichment of the 186 TCF7L2 target genes that showed bipolar disorder as the only significant enrichment in the Jensen’s Lab Disease database. No other pathway was significantly (FDR < 0.01) enriched in databases on the Enrichr website. **b** Expression levels of BD risk genes enriched in the disease pathway analysis were significantly changed after TCF7L2 KD in hiPSC-derived astrocytes. **c** Integrative Genomic Views of TCF7L2 ChIP peaks mapped to the BD risk genes shown in **b**. The orange panel represents the input control. The green panel represents TCF7L2 ChIP-seq reads. The purple panel represents TCF7L2 ChIP peaks that were called based on statistical significance (FDR < 0.01). Major ChIP peaks have been highlighted in dotted boxes. Genes mapping to the TCF7L2 ChIP peaks are shown in the blue panels at the bottom of each panel. The names of BD risk genes for which expression was changed after TCF7L2 KD are highlighted in red.

To further investigate possible disease pathway(s) that might be related to the 186 TCF7L2 target genes in astrocytes, disease pathway enrichment was performed. BD was the most significantly enriched disease for these genes (FDR < 0.05) based on the Jensen disease database [69] (Fig. 5a). Eight of the enriched BD genes were *NCAN*, *TENM4* (previously named *ODZ4*), *FBLN1*, *ZMIZ1*, *NFIA*, *ZSWIM6*, *DOK5,* and *SLC45A4*. Expression of all eight of these BD risk genes was significantly changed in astrocytes after TCF7L2 KD (Fig. 5b). Finally, ChIP-seq demonstrated that TCF7L2 bound directly to sequences in or near all of these genes, suggesting direct regulation of their expression (Fig. 5c). Integration of RNA-seq and ChIP-seq improved the accuracy of the identification of TCF7L2 target genes, e.g., TCF7L2 ChIP peaks mapped ~100 kb away from *NCAN* and *SCL45A4* (Fig. 5c) but based on the RNA-seq data, no other genes that mapped nearer to those peaks were differentially expressed in hiPSC-derived astrocytes after TCF7L2 KD (Supplementary Table S7), suggesting possible direct regulation of *NCAN* and *SCL45A4* expression. Seven of the TCF7L2-regulated BD risk genes shown in Fig. 5b, with the exception of *SCL45A4,* which encodes a sucrose transporter, are known to function in CNS cellular differentiation or neurodevelopment (Supplementary Table S8). Furthermore, seven of those eight genes (all except *TENM4*) contain genetic polymorphisms that are genome-wide significantly associated with either BMI or metabolic phenotypes (see Supplementary Table S8). These results suggested that TCF7L2 may directly regulate genes associated with BD risk and BMI or metabolic phenotypes in astrocytes, a possible molecular mechanism related to the role of *TCF7L2* in the comorbidity of BD and obesity.

## Discussion

Metabolic comorbidities are highly prevalent in BD and are associated with more severe mood symptoms and worse treatment outcomes [16–19]. Disrupted insulin signaling in the brain and the hypothalamic–pituitary–adrenal axis has been proposed as one explanation for the comorbidity of mood disorders and obesity [70]. Recent large GWA studies of BD risk [6, 7] and obesity [71] have provided additional evidence for genetic correlations between BD and metabolic syndromes. Specifically, pathway enrichment of GWAS-identified BD risk genes often identified metabolic pathways such as insulin signaling and energy metabolism [6, 7]. Moreover, pathway analyses of BMI-related genes have strongly supported a role for the CNS in obesity susceptibility [71]. Obesity management by pharmacologic treatment in patients with psychiatric disorders is presently being tested as a novel therapeutic intervention [72]. In an effort to understand the possible contribution of genetics to the comorbidity of BD and obesity, we previously performed a GWAS for BD risk incorporating interaction with BMI, a study in which we identified a genome-wide significant SNP (rs12772424) associated with BMI-related BD that mapped to the *TCF7L2* gene [20]. In the present study, we functionally characterized *TCF7L2* in astrocytes, CNS cells that highly express *TCF7L2* (Fig. 1), in an attempt to understand its possible role in the comorbidity of BD and obesity. Our results suggest that the *TCF7L2* gene might play a role in both CNS development and in the regulation of metabolism in astrocytes, at least in part through the actions of different TCF7L2 variant transcripts.

TCF7L2 has been studied extensively as a TF in peripheral tissues with a role in glucose metabolism and T2D risk. The TCF7L2 protein has also been well characterized and includes a β-catenin-binding domain, suggesting a role in the canonical Wnt pathway (or Wnt/β-catenin pathway) [73]. Our studies have confirmed its role in Wnt signaling in hiPSC-derived astrocytes (Fig. 4), a function that might be related to BD risk (see Fig. 5). However, *TCF7L2* transcription is complicated, e.g., with a total of 30 transcript variants listed in the *Ensembl* database. More than half of those transcript variants do not include exons 1–4 (Fig. 2a), which encode the β-catenin-binding domain [73], supporting Wnt-independent *TCF7L2* functions that have been observed in the mouse brain [38]. The *TCF7L2* rs12772424 SNP that we reported led us to a series of lncRNA-TCF7L2 transcripts that do not include exons 1–4, and specifically to “T-3”, which is highly expressed in the human brain and with an expression that is regulated by glucocorticoid signaling (Fig. 2). LncRNAs are known to affect the expression of a large number of genes by both *cis*- and *trans*-regulation [74] and to play key roles in various brain disorders [75]. Our functional characterization of “T-3” in hiPSC-derived astrocytes revealed a role in the regulation of the expression of genes involved in diabetes and insulin signaling pathways (Fig. 3), results which suggest that in the CNS *TCF7L2*, in addition to its role in the liver [32–34], pancreas [35, 36], and gut [37], might also contribute to metabolic regulation.

Abnormal cortisol levels in BD patients have been observed during DEX/CRH testing [76] and the GR is expressed in astrocytes [77]. Astrocytes have recently been recognized as key players in neuropsychiatric disorders, including BD [78, 79], and they have been proposed as cellular targets for BD pharmacotherapy [80]. Recent studies have demonstrated that dysfunction of insulin signaling in hypothalamic astrocytes can influence systemic glucose levels in mice [56, 57]. Of course, these results with experimental animals will also have to be pursued in human subjects. Our identification of the “T-3” glucocorticoid-responsive lncRNA*-*TCF7L2 (Fig. 2), a non-coding transcript that regulates the expression of both TCF7L2 mRNA and of genes involved in insulin signaling pathways in hiPSC-derived astrocytes (Fig. 3) may help to explain, in part, the correlation of glucocorticoid signaling, insulin signaling, and BMI in BD.

Finally, we should point out the limitations of our studies, beginning with our use of cell line models. We used hiPSC-derived astrocytes to study *TCF7L2* molecular function because *TCF7L2* is more highly expressed in astrocytes than in other CNS cell types and, to our knowledge, its function in human CNS cell model(s) has not been reported previously. However, *TCF7L2* is also expressed in neurons and oligodendrocyte progenitor cells (see Fig. 1), and it may regulate oligodendrocyte differentiation in mice [38, 39]. Whether *TCF7L2* in other CNS cell types might contribute to BD and/or BMI is unknown and should be the subject of future studies. However, our data strongly suggest that *TCF7L2* may play dual roles in the regulation of astrocytic insulin signaling and CNS development, perhaps through the effects of different RNA transcripts, a possibility that should also be pursued in the future. Obviously, molecular mechanisms based on cell line model systems are not able to provide a comprehensive view of physiological processes underlying clinical phenotypes. Therefore, genetically modified animal models and/or three-dimensional human brain organoids represent logical next steps in pursuit of the observations reported here. We should also point out that, although BMI was the best obesity-related phenotype that we had for all of our GWAS patients, we appreciate that it is not an ideal measure of obesity [21] and that other phenotypes such as waist circumference could provide complementary information on obesity-related phenotypes. In this context, it is of interest that “waist circumference adjusted for BMI” emerged as a top GWAS Catalog phenotype in our pathway enrichment analysis using DEGs after knockdown of the lncRNA-TCF7L2 transcript in astrocytes (see Fig. 3j).

In summary, by setting out to define the molecular function of *TCF7L2* in hiPSC-derived astrocytes, we have demonstrated that glucocorticoid regulation of the transcription of a *TCF7L2* encoded non-coding transcript in a SNP-dependent fashion might influence astrocytic insulin signaling pathways, an effect which could also influence systemic glucose metabolism. We also obtained evidence that astrocytic TCF7L2 might contribute to BD pathophysiology by regulating the expression of BD risk genes. These results strongly support the possibility that the function of *TCF7L2*, and specifically the function of the lncRNA-TCF7L2 T-3 variant transcript in the CNS might represent an important link between BD, BMI, and metabolism.

## Supplementary information

Supplementary Text

Supplementary Figures

Supplementary Tables

Supplementary Table S6

Supplementary Table S7

## Data Availability

The RNA-seq and ChIP-seq data sets generated during the current study are available in the Gene Expression Omnibus with accession ID: GSE179922.
