# Supplementary material for: TCF7L2 lncRNA: a link between bipolar disorder and body mass index through glucocorticoid signaling
Source: Mol Psychiatry. 2021 Sep 17;26(12):7454–64. doi: 10.1038/s41380-021-01274-z (PMC8872993; doi:10.1038/s41380-021-01274-z)
Supplement: Supplementary file 1 — Supplementary Text [file 41380_2021_1274_MOESM1_ESM.docx]

**Supplementary Text**

**Materials and Methods**

***TCF7L2* Transcription in the Human Brain at the Single Cell Level**

To understand *TCF7L2* transcription in the human brain at single cell level, we first consulted single-nucleus RNA-seq (snRNA-seq) datasets generated by the Allen Institute (<https://portal.brain-map.org/atlases-and-data/rnaseq>)^1^. Their dataset included single-nucleus transcriptomes from 49,495 nuclei across multiple human cortical areas, including the middle temporal gyrus (MTG), anterior cingulate cortex, primary visual cortex, primary motor cortex, primary somatosensory cortex, and primary auditory cortex. Nuclei were dissociated and sorted using the neuronal marker NeuN. Nuclei were sampled from post-mortem and neurosurgical (MTG only) donor brains and transcriptomes were profiled with SMART-Seq v4 RNA-sequencing. We then combined and analyzed two additional single-cell RNA-seq (scRNA-seq) datasets as described previously^2^. Briefly, two scRNA-seq datasets, one that included 2498 glioblastoma adjacent cells identified as non-neoplastic from the cortex of four patients (GEO accession: GSE84465)^3^ and the other that included 466 cells from brain cortical tissue removed from eight individuals with medically refractory seizures (GEO accession: GSE67835)^4^, were downloaded from SRA, mapped to the human reference genome GRCh38/hg38 using STAR^5^, and processed by use of the pagoda2 package^6^. Raw counts were called by HTSEQ^7^. Cells with fewer than 10^5^ total counts were excluded from further analysis. Cell types were identified by the UMAP with the umapr package^8^. Finally, chromatin accessibility data for the *TCF7L2* gene in human brain cells were obtained from a single-cell assay for transposase-accessible chromatin a using sequencing (scATAC-seq) dataset that included 70,631 cells from 10 different samples across human brain regions^9^. The scATAC-seq data was visualized by using the WashU Epigenome Browser^10^.

**Identification of Glucocorticoid Response Elements (GREs)**

The *TCF7L2* rs12772424 SNP was annotated using HaploReg v4.1^11^ which identified the fact that the rs12772424 variant allele (A) created a GRE motif. A motif scanning tool, Find Individual Motif Occurrences (FIMO) (<http://meme-suite.org/tools/fimo>)^12^, was then applied to identify sequences matched to known GRE motifs across the rs12772424 SNP locus. DNA sequences of the known GRE motif, “AGAACA”, was used as input for putative GRE scanning. Based on linkage disequilibrium (LD) and haplotype information for the rs12772424 SNP in a European population, DNA sequences mapped ±8.5 Kb of the rs12772424 SNP were scanned for GRE motifs using FIMO. Chromatin-immunoprecipitation sequencing (ChIP-seq) data generated in cell lines treated with the glucocorticoid receptor agonist, dexamethasone, were obtained from the ENCODE project^13^.

**Cell Culture**

A human induced pluripotent stem cell (hiPSC)-derived astrocyte progenitor kit (cat#: ax0081; discontinued) was purchased from Axol Bioscience Ltd. (Cambridge, United Kingdom), and hiPSC-derived hepatocytes (Cellartis® Enhanced hiPS-HEPv2 from ChiPSC12, cat#: Y10133) and pancreatic β-cells (Cellartis® hiPS Beta Cells from ChiPSC12, cat#: Y10100) were purchased from Takara Bio Inc. (Mountain View, CA, USA). Cells were cultured by following the manufacturers’ protocols. Mature cells were characterized by immune staining of cell specific markers. Specifically, the hiPSC-derived astrocyte progenitors were seeded in plates coated with Matrigel™ hESC-Qualified Matrix (cat#:354277; Corning Life Sciences). Cells matured during 17 days of culture in Astrocyte Maintenance Medium, which consisted of Neurobasal™-A medium (cat#:12349015, ThermoFisher), 2% (v/v) fetal bovine serum (FBS) (cat#:A3160401, ThermoFisher), 1% (v/v) GlutaMAX (cat#:35050061, ThermoFisher), 2% (v/v) N21-Max (cat#: AR008, R&D Systems), 10 ng/mL of heregulin-β1 (cat#:100-03, Peprotech) and 8 ng/mL of fibroblast growth factor 2 (FGF2) (cat#:ax0047, Axol Bioscience). Mature astrocytes were then aliquoted and stored in liquid nitrogen until used. Cells that were used in all experiments had fewer than 5 passages. The hiPSC-derived hepatocytes were cultured for 19 days after thawing until they were used. The hiPSC-derived β-cells were cultured for 15 days. Before use, all three hiPSC-derived cell lines were characterized by immune staining of cell specific makers as described subsequently. The A549 human lung carcinoma cell line was obtained from the ATCC and cultured in F-12K medium (cat#: 30-2004, ATCC) supplemented with 10% FBS. The U-251 MG human glioblastoma cell line was obtained from Sigma (cat#: 09063001) and cultured in DMEM/F-12 medium (cat#:11320033, ThermoFisher) supplemented with 10% FBS.

**Immunofluorescent Staining**

Mature hiPSC-derived astrocytes, hepatocytes and β-cells were grown in 16-well CultureWell™ Chambered Coverglass (cat#:C37000, ThermoFisher) for all immune-fluorescence assays. Specifically, cells were washed with PBS once before fixation with 4% methanol-free formaldehyde (cat#: 28906, ThermoFisher). After fixation, cells were subjected to Trixton-X100 0.1% treatment before immunostaining. Fixed cells were then blocked with 1% BSA solution, followed by overnight incubation with primary antibodies diluted in 1% BSA at 4 °C. For astrocytes, primary antibodies against glial fibrillary acidic protein (GFAP) (cat#: 3670S, Cell Signaling) at a 1:300 dilution, and against S100 calcium binding protein beta (S100β) (cat#: ab52642, Abcam) at a 1:100 dilution, were added. For hepatocytes, primary antibodies against albumin (ALB) (cat#: GTX102419, GeneTex) at a 1:400 dilution, and against hepatocyte nuclear factor 4 alpha (HNF4α) (cat#: MAB4605, R&D Systems) at 10 µg/mL, were used. For pancreatic β-cells, primary antibodies against insulin (INS) (cat#: I2018-100UL, Sigma) at a 1:1000 dilution, and against MAF BZIP transcription factor A (MAFA) (cat#: 79737S, Cell Signaling) at a 1:1000 dilution, were used. Cells were then washed 3 times with PBS before being exposed to the secondary antibodies, goat-anti-mouse IgG conjugated with Alexa Fluor® 488 (cat#: 4408S, Cell Signaling) and goat-anti-rabbit IgG conjugated with Alexa Fluor® 594 (cat#: 8889S, Cell Signaling), for 1 hour at room temperature. After 3 PBS washes, mounting solution with DAPI was added to the cells. Photos of astrocytes and hepatocytes stained with these markers were then taken using a Zeiss LSM 780 confocal microscope.

**Dexamethasone (DEX) Treatment**

For “screening” of TCF7L2 variant transcript expression after the activation of glucocorticoid signaling, three cell lines, including hiPSC-derived astrocytes, A549 lung carcinoma cells and U-251 MG glioblastoma cells, were treated with 100 nM of DEX (cat#: D2915, Sigma) for 6 hours. Before DEX treatment, mature hiPSC-derived astrocytes were maintained in Astrocyte Maintenance Medium with N-2 Supplement (cat#:17502048, ThermoFisher) instead of N21-MAX media Supplement, which contains corticosterone, for 48 hours. A549 and U-251 MG cells were maintained in media supplimented with 5% charcoal-stripped FBS for 24 hours. After 6 hours of DEX or vehicle (Veh) treatment, cells were harvested for total RNA extraction and TCF7L2 RNA levels were measured by qRT-PCR as described subsequently. In the DEX dose-dependent assays, cells were treated with DEX at 100 nM, 10 nM and 1 nM for 6 hours.

**Quantitative Reverse Transcription Polymerase Chain Reaction (qRT-PCR)**

Total RNA was extracted using the QIAGEN miRNeasy Mini Kit (cat#: 217004) according to the manufacturer’s instructions. Primers targeting TCF7L2 exon junctions (**Supplementary Table S4**) were used to differentiate and quantify TCF7L2 transcript variants. Power SYBR™ Green RNA-to-CT™ 1-Step Kit (cat#: 4389986, ThermoFisher) was used for qRT-PCR. For each reaction, 100 ng of total RNA, 0.2 µM of forward primers plus 0.2 µM reverse primers were mixed in a total volume of 10 µL reaction. PCR was conducted with a QuantStudio 5 platform (ThermoFisher). Gene expression analyses were performed using the ΔΔCt method. For cell lines treated with DEX, expression of a glucocorticoid-inducible gene, *FKBP5*, was determined as a treatment control, and *GAPDH* was used as the internal reference gene. Total RNA samples from pooled (from 1 to 18 adults) human brain (cat#: 636530, lot#: 1711057), liver (cat#: 636531, lot#: 1703002), pancreas (cat#: 636577, lot#: 2009171A), stomach (cat#: 636578, lot#: 1904758A), small intestine (cat#:636539, lot#: 1611206A), and colon (cat#: 636553, lot#: 1804052) tissues were obtained from Takara Bio. These total RNA samples were used for the quantification of *TCF7L2* transcript variants by qRT-PCR. The *VCP* and *C1orf43* genes were used as housekeeping genes since their RNA levels are more consistent across human tissues than are commonly used housekeeping genes, such as *GAPDH*, for which expression has been found to vary considerably across different tissues^14^.

**Reporter Gene Assay**

To determine the effects of the rs12772424 SNP on TCF7L2-lncRNA transcription, a 373 base pair (bp) DNA segment that included the rs12772424 SNP in tandem with an approximately 1.8-kb DNA segment that included TCF7L2 exons 4b-4e sequence was subcloned upstream of the *Luc2* gene (encoding firefly luciferase) in the pGL4.23 vector (cat#: E8411, Promega). DNA sequences of the primers used for subcloning the rs12772424 SNP-containing DNA fragment were 5′-caatGAGCTCTACTGAGTTGCACCGAGCAC-3′ (forward primer with the SacI restriction enzyme site underlined) and 5′-caatCTCGAGTGACCATACTCCCTCTTTGCTT-3′ (reverse primer with the XhoI restriction enzyme site underlined). DNA sequences of the primers used for subcloning the TCF7L2 exons 4b-4e DNA fragment were 5′-caatCTCGAGCGAATCGCTCGTTCTCTGTT-3′ (forward primer with the XhoI restriction enzyme site underlined) and 5′-cgacAAGCTTAACATGGCAAAGGCTGAGAG-3′ (reverse primer with the HindIII restriction enzyme site underlined). Genomic DNA from lymphoblastoid B-cell lines (LCLs) with known genotypes^15^ for the SNP of interest was used as template for PCR reactions to amplify DNA fragments containing wild-type (WT) or variant (V) SNP alleles. After confirmation of the sequence by DNA sequencing, reporter gene constructs containing WT or V SNP genotypes were transfected into cells using Lipofectamine™ 3000 Reagent (ThermoFisher). A pRL-TK vector that expresses renilla luciferase (cat#: E2241, Promega) was co-transfected as an internal control. After DEX treatment, the cells were lysed, and luciferase activity was determined using the Dual-Luciferase® Reporter Assay System kit (cat#: E1910, Promega).

**TCF7L2 cDNA Amplification**

A total RNA sample from pooled (from 5 adults) human brain (cat#: 636530, lot#: 2006022, Takara Bio.) and total RNA extracted from hiPSC-derived astrocytes were used as template to amplify TCF7L2 cDNA using RT-PCR. TCF7L2 gene-specific primers, including their mapping exons and primer combinations used for PCR are shown in **Fig. 3.** Primer sequences are listed in **Supplementary Table S5**. RT-PCR was performed using the SuperScript™ IV One-Step RT-PCR System (cat#: 12595025, ThermoFisher). For each RT-PCR, 200ng of human brain total RNA was used. A human brain Marathon®-ready cDNA library (cat#: 639300, Takara Bio) was also used as template to amplify TCF7L2 cDNA. Marathon®-Ready cDNA was made from high-quality poly(A)+ RNA using a procedure optimized to produce full-length cDNA and to eliminate 3' heterogeneity. After cDNA synthesis, blunt ends were created and the Marathon Adaptor was ligated to both ends of the double-stranded cDNA. The same sets of TCF7L2 gene-specific primers (see **Fig. 3a** and **Supplementary Table S5**), and the Advantage® 2 PCR Kit (cat#: 639207, Takara Bio) were used to amplify TCF7L2 cDNA when the poly(A)+ cDNA library was used as PCR template. PCR products were run on a 1.2% agarose gel for visualization.

**Transient Transfection for TCF7L2 Knock-down**

To knock-down (KD) the lncRNA-TCF7L2 “T-3” in hiPSC-derived astrocytes, the cells were transfected with two LNA GapmeRs antisense oligonucleotides (ASO) (ASO1 cat#: 339511 LG00226568-DDA; ASO2 cat#: 339511 LG00226571-DDA, QIAGEN) targeted to TCF7L2 exon-4d, the unique exon of “T-3”, using Lipofectamine™ RNAiMAX Reagent (ThermoFisher). Cells transfected with Negative control B Antisense LNA GapmeR ASO (cat#: 339515 LG00000001-DDA, QIAGEN) were treated as non-targeting control. To KD TCF7L2 mRNA in hiPSC-derived astrocytes, cells were transfected with siGENOME™ SMARTpool siRNAs targeting TCF7L2 exons 5 and 14 (cat#: M-003816-02-0005, Dharmacon) using Lipofectamine™ RNAiMAX Reagent (ThermoFisher). Cells were also transfected with non-targeting siRNAs (cat#: D-001206-13-05, Dharmacon) as a control. Cells were harvested for the extraction of total RNA and protein after 24 and 48 hours of transfection, respectively.

**Western Blot Assay**

Total protein lysates from cultured cells were prepared by adding Lysis Buffer (25 mM Tris-HCl pH 7.4, 150 mM NaCl, 1 mM EDTA, 1% NP-40 and 5% glycerol), followed by centrifugation at 14,000×g for 10 mins. Protein concentrations were determined by using the Pierce BCA Protein Assay Kit (cat#:23227, ThermoFisher). Equal quantities of denatured protein were loaded onto 4–20% Mini-PROTEAN® TGX™ Precast Protein Gels (Bio-Rad, Hercules, CA) to separate proteins. Proteins were transferred electrophoretically from the gels to PVDF membranes (Bio-Rad, Hercules, CA) which were then blocked with 5% non-fat milk at room temperature for 1 hour. After washing with TBST, membranes were incubated with anti-TCF7L2 antibody (cat#:2569S, Cell Signaling; 1:1000 dilute) or anti-Vinculin antibody (cat#:V9264, Sigma; 1:5000 dilute), which were dissolved in 1% BSA prepared in TBST at 4°C overnight with gentle rocking. Following incubation, the membranes were washed vigorously three times in TBST buffer and were then incubated with horseradish peroxidase (HRP)-labelled secondary antibody which was dissolved in 5% non-fat milk at room temperature for 1 hour. The SuperSignal West Dura Extended Duration Substrate (cat#:34075, ThermoFisher), a luminol-based enhanced chemiluminescence HRP substrate, was applied to the membranes, and radiographic images were captured by use of the ChemiDoc™ Touch Image System (Bio-Rad, Hercules, CA). Vinculin protein was employed as a loading control.

**RNA sequencing (RNA-seq)**

Total RNA was extracted with TRIzol® Reagent (ThermoFisher) and the miRNAeasy kit (QIAGEN) per manufacturer’s instructions. RNA quality control was performed before RNA-seq, which showed that the RNA integrity number (RIN) was ≥ 9 for all samples. RNA-seq libraries were generated using the Illumina TruSeq RNA Library Prep Kit v2 (Illumina, San Diego, CA). Paired-end sequencing 2×100bp was performed on an Illumina HiSeq 4000 with approximately 50 million fragment reads per sample. Each sample was sequenced in duplicate. RNA sequencing quality was determined with FastQC (Babraham Institute, Cambridge, UK), and the reads were aligned to hg38 using STAR^5^. Raw counts were generated with featureCounts^16^. Downstream differential expression analysis was conducted with the EdgeR package^17^ using R. Significantly differentially expressed genes (DEGs) were defined as having an FDR < 0.05, and fold change of ≥ 2.0. DEGs were then subjected to pathway analysis using EnrichR^18^, and Gene Ontology^19^.

**Chromatin-immunoprecipitation Sequencing (ChIP-seq)**

For mature hiPSC-astrocytes, 20 million cells were fixed with 1% methanol-free formaldehyde (ThermoFisher). The reaction was stopped by adding 0.125 M glycine for 5 minutes at room temperature. Cell pellets were washed with ice-cold Tris-buffered saline (TBS), and sample preparation for ChIP-seq was performed as described by *Zhong et al.*, 2017^20^. A total volume of 16 µL TCF7L2 antibody (cat#: 2569S, Lot 4, Cell Signaling) was used to precipitate TCF7L2-DNA complexes per reaction. After library preparation, paired-end sequencing was performed with an Illumina Highseq 4000. Raw sequencing reads were processed and analyzed using the HiChIP pipeline^21^ to obtain integrative genomics viewer files and a list of peaks. Visualization of the ChIP-seq result was conducted using deepTools software^22^. Integration of TCF7L2 ChIP-seq and RNA-seq to map peaks to differentially-expressed genes after TCF7L2 KD were conducted with the binding and expression target analysis (BETA)^23^ software with parameters –d 100000 –da 500 –df 0.05.

**References:**

1. Hodge RD, Bakken TE, Miller JA, Smith KA, Barkan ER, Graybuck LT *et al.* Conserved cell types with divergent features in human versus mouse cortex. *Nature* 2019; **573**(7772)**:** 61-68.

2. Liu D, Zhuang Y, Zhang L, Gao H, Neavin D, Carrillo-Roa T *et al.* ERICH3: vesicular association and antidepressant treatment response. *Mol Psychiatry* 2020.

3. Darmanis S, Sloan SA, Croote D, Mignardi M, Chernikova S, Samghababi P *et al.* Single-Cell RNA-Seq Analysis of Infiltrating Neoplastic Cells at the Migrating Front of Human Glioblastoma. *Cell Rep* 2017; **21**(5)**:** 1399-1410.

4. Darmanis S, Sloan SA, Zhang Y, Enge M, Caneda C, Shuer LM *et al.* A survey of human brain transcriptome diversity at the single cell level. *Proc Natl Acad Sci U S A* 2015; **112**(23)**:** 7285-7290.

5. Dobin A, Davis CA, Schlesinger F, Drenkow J, Zaleski C, Jha S *et al.* STAR: ultrafast universal RNA-seq aligner. *Bioinformatics* 2013; **29**(1)**:** 15-21.

6. Kharchenko PV, Silberstein L, Scadden DT. Bayesian approach to single-cell differential expression analysis. *Nature Methods* 2014; **11**(7)**:** 740-742.

7. Anders S, Pyl PT, Huber W. HTSeq--a Python framework to work with high-throughput sequencing data. *Bioinformatics* 2015; **31**(2)**:** 166-169.

8. Becht E, McInnes L, Healy J, Dutertre C-A, Kwok IWH, Ng LG *et al.* Dimensionality reduction for visualizing single-cell data using UMAP. *Nat Biotechnol* 2018; **37:** 38.

9. Corces MR, Shcherbina A, Kundu S, Gloudemans MJ, Fresard L, Granja JM *et al.* Single-cell epigenomic analyses implicate candidate causal variants at inherited risk loci for Alzheimer's and Parkinson's diseases. *Nat Genet* 2020; **52**(11)**:** 1158-1168.

10. Zhou X, Lowdon RF, Li D, Lawson HA, Madden PA, Costello JF *et al.* Exploring long-range genome interactions using the WashU Epigenome Browser. *Nat Methods* 2013; **10**(5)**:** 375-376.

11. Ward LD, Kellis M. HaploReg v4: systematic mining of putative causal variants, cell types, regulators and target genes for human complex traits and disease. *Nucleic Acids Res* 2016; **44**(D1)**:** D877-881.

12. Grant CE, Bailey TL, Noble WS. FIMO: scanning for occurrences of a given motif. *Bioinformatics* 2011; **27**(7)**:** 1017-1018.

13. Davis CA, Hitz BC, Sloan CA, Chan ET, Davidson JM, Gabdank I *et al.* The Encyclopedia of DNA elements (ENCODE): data portal update. *Nucleic Acids Res* 2018; **46**(D1)**:** D794-D801.

14. Eisenberg E, Levanon EY. Human housekeeping genes, revisited. *Trends Genet* 2013; **29**(10)**:** 569-574.

15. Niu N, Qin Y, Fridley BL, Hou J, Kalari KR, Zhu M *et al.* Radiation pharmacogenomics: a genome-wide association approach to identify radiation response biomarkers using human lymphoblastoid cell lines. *Genome Res* 2010; **20**(11)**:** 1482-1492.

16. Liao Y, Smyth GK, Shi W. featureCounts: an efficient general purpose program for assigning sequence reads to genomic features. *Bioinformatics* 2014; **30**(7)**:** 923-930.

17. Robinson MD, McCarthy DJ, Smyth GK. edgeR: a Bioconductor package for differential expression analysis of digital gene expression data. *Bioinformatics* 2010; **26**(1)**:** 139-140.

18. Kuleshov MV, Jones MR, Rouillard AD, Fernandez NF, Duan Q, Wang Z *et al.* Enrichr: a comprehensive gene set enrichment analysis web server 2016 update. *Nucleic Acids Res* 2016; **44**(W1)**:** W90-97.

19. Ashburner M, Ball CA, Blake JA, Botstein D, Butler H, Cherry JM *et al.* Gene ontology: tool for the unification of biology. The Gene Ontology Consortium. *Nat Genet* 2000; **25**(1)**:** 25-29.

20. Zhong J, Ye Z, Lenz SW, Clark CR, Bharucha A, Farrugia G *et al.* Purification of nanogram-range immunoprecipitated DNA in ChIP-seq application. *BMC Genomics* 2017; **18**(1)**:** 985.

21. Yan H, Evans J, Kalmbach M, Moore R, Middha S, Luban S *et al.* HiChIP: a high-throughput pipeline for integrative analysis of ChIP-Seq data. *BMC Bioinformatics* 2014; **15:** 280.

22. Ramirez F, Ryan DP, Gruning B, Bhardwaj V, Kilpert F, Richter AS *et al.* deepTools2: a next generation web server for deep-sequencing data analysis. *Nucleic Acids Res* 2016; **44**(W1)**:** W160-165.

23. Wang S, Sun H, Ma J, Zang C, Wang C, Wang J *et al.* Target analysis by integration of transcriptome and ChIP-seq data with BETA. *Nat Protoc* 2013; **8**(12)**:** 2502-2515.
