# Supplementary material for: TCF7L2 lncRNA: a link between bipolar disorder and body mass index through glucocorticoid signaling
Source: Mol Psychiatry. 2021 Sep 17;26(12):7454–64. doi: 10.1038/s41380-021-01274-z (PMC8872993; doi:10.1038/s41380-021-01274-z)

**Supplementary Figures S1-S4**

**Supplementary Figure S1. *TCF7L2* Expression in Human Brian at the Single Cell Level.** Analysis of single-cell RNA-seq (scRNA-seq) data generated from surgically-removed human cerebral cortical tissues in two studies^41, 42^. (**a**) Clusters of human brain cells were assigned cell types based on the expression of cell-type-specific marker genes quantified by scRNA-seq from 2,770 cells. Each dot represents a single cell. Cell types have been color-coded. (**b**) TCF7L2-positive cells were color-coded based on TCF7L2 expression levels, and the TCF7L2-negative cells are shown as gray dots. For astrocytes, the percentage refers to cells positive for TCF7L2.


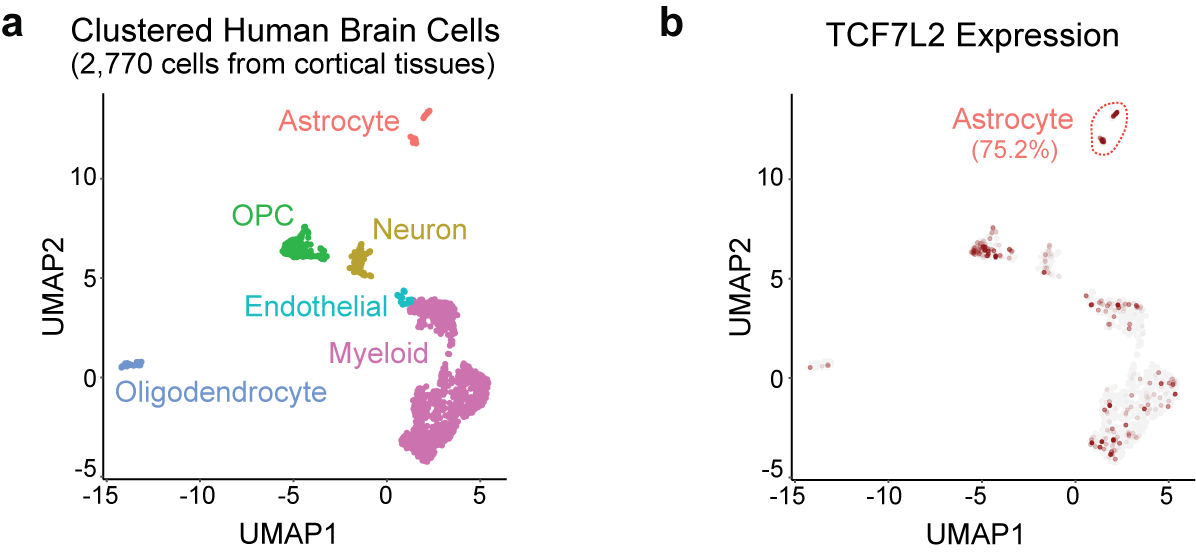


**Supplementary Figure S2. *TCF7L2* transcript variant expression can be regulated by glucocorticoids.** (**a**) *TCF7L2* transcript variant expression in A549 human lung carcinoma cells after treatment with dexamethasone (DEX), 100 nM for 6 hours. RNA levels were determined by qRT-PCR using primers targeting *TCF7L2* exon junctions (“J”) as shown on the x-axis. Exon numbers are as labelled in **Fig. 2a**. *GAPDH* expression was measured as an internal control. The RNA level of *FKBP5*, a prototypic glucocorticoid induced gene, was quantified as a control for DEX treatment. The y-axis represents log_2_ fold change (FC) of RNA levels after DEX treatment when normalized to vehicle treatment (DEX/Veh). Data are mean ± s.d. (n=3), with statistical significance determined by Dunnett’s test denoted as ****P* <0.001. RNA levels for *TCF7L2* exon junctions J4b-5 (T-2), J4d-5 (T-3) and J4e-5, were significantly decreased after DEX treatment. Other TCF7L2 exon junctions were neither significantly changed after DEX treatment nor detectable by qRT-PCR (data not shown). (**b**) DEX-dose dependent induction of FKBP5 (left) and repression of TCF7L2 “T-2” and “T-3” (middle and right) in A549 cells. (**c**) Repression of *TCF7L2* exons junctions J4b-5 (T-2) and J4d-5 (T-3), after 100 nM DEX treatment was also observed in U-251 MG glioblastoma cells. *TCF7L2* exon junction J4e-5 was not detectable, indicating that exon 4e was not expressed in this cell line, similar to the situation in hiPSC-derived astrocytes **(Fig. 2e**). (**d**) DEX-dose dependent induction of FKBP5 (left) and repression of TCF7L2 “T-2” and “T-3” (middle and right) in U-251 MG cells.


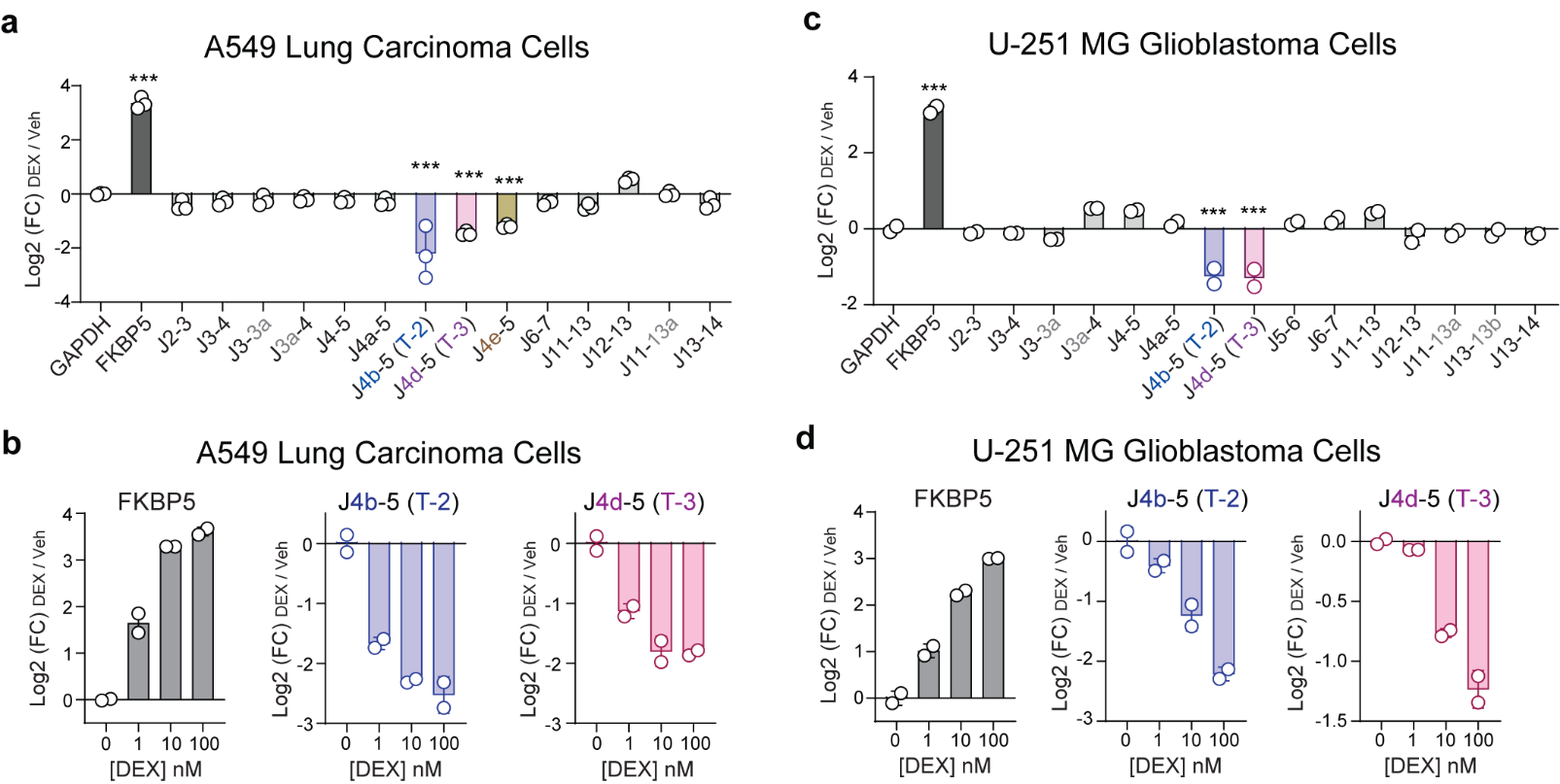


**Supplementary Figure S3. Characterizations of Human iPSC-derived Hepatocytes and Pancreatic β-Cells.** Immunofluorescent staining of (**a**) hepatocyte markers, hepatocyte nuclear factor 4 alpha (HNF4α) and albumin (ALB), in iPSC-derived hepatocytes, and of (**b**) pancreatic β-cell markers, insulin (INS) and MAF BZIP transcription factor A (MAFA), in iPSC-derived pancreatic β-cells. Scale bars represent 20 µM in all photographs.


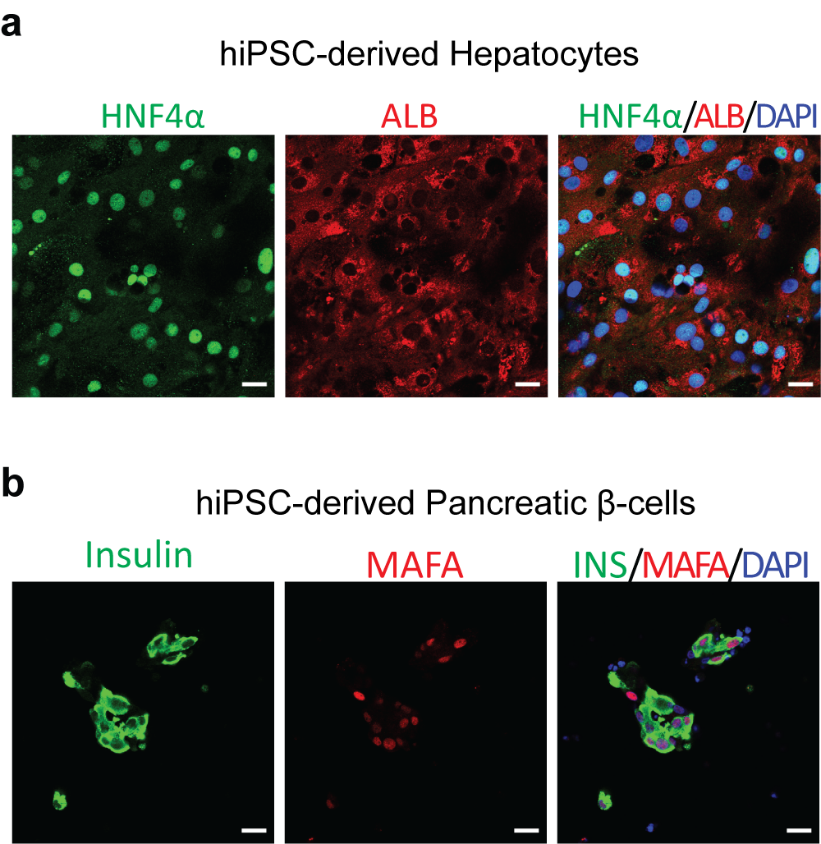


**Supplementary Figure S4. Western Blot Assays for TCF7L2.** TCF7L2 proteins were blotted in (**a**) the U251-MG glioblastoma cells and (**b**) in A549 lung carcinoma cells after knock-down of the lncRNA-TCF7L2 “T-3” using “pooled” ASOs 1 and 2. Control samples were cells transfected with non-targeting ASOs. Vinculin was blotted as an internal protein control.


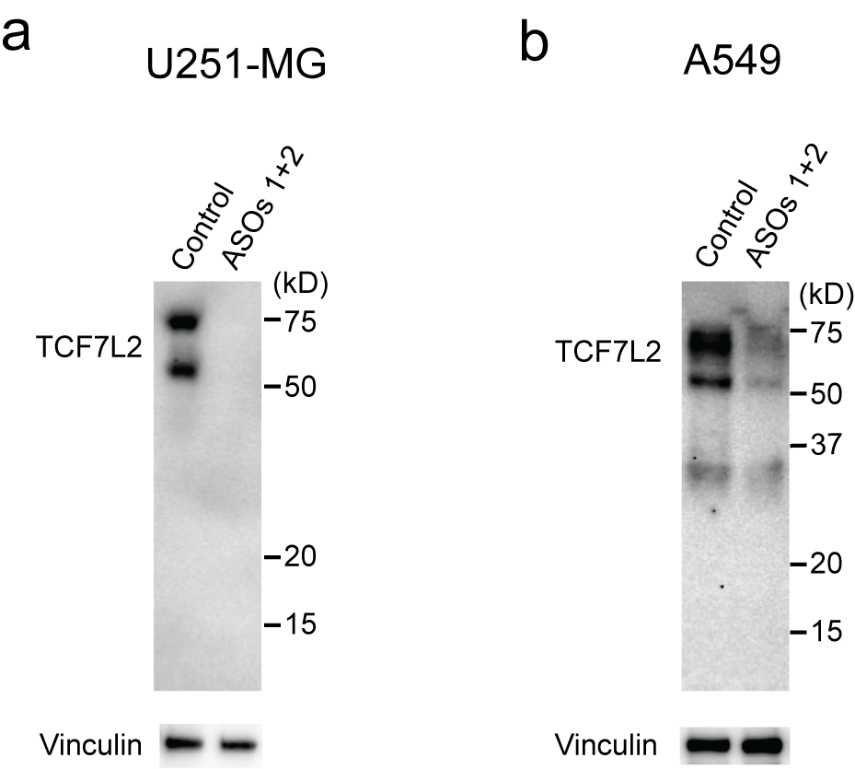

Supplement: Supplementary file 2 — Supplementary Figures [file 41380_2021_1274_MOESM2_ESM.docx]
