# Supplementary material for: TCF7L2 lncRNA: a link between bipolar disorder and body mass index through glucocorticoid signaling
Source: Mol Psychiatry. 2021 Sep 17;26(12):7454–64. doi: 10.1038/s41380-021-01274-z (PMC8872993; doi:10.1038/s41380-021-01274-z)
Supplement: Supplementary file 3 — Supplementary Tables [file 41380_2021_1274_MOESM3_ESM.docx]

**Supplementary Tables S1-S8**

**Table S1. Key Resources.**

| **REAGENT or RESOURCE** | **SOURCE** | **CATALOG NUMBER or IDENTIFIER** |
| --- | --- | --- |
| **Antibodies** |  |  |
| Anti-TCF7L2 | Cell Signaling | 2569S |
| Anti-Vinculin | Sigma | V9264 |
| Anti-GFAP | Cell Signaling | 3670S |
| Anti-S100β | Abcam | ab52642 |
| Anti-Insulin (INS) | Sigma | I2018-100UL |
| Anti-MAFA | Cell Signaling | 79737S |
| Anti-albumin (ALB) | GeneTex | GTX102419 |
| Anti-HNF4α | R&D Systems | MAB4605 |
| Anti-mouse IgG (Alexa Fluor® 488) | Cell Signaling | 4408S |
| Anti-rabbit IgG (Alexa Fluor® 594) | Cell Signaling | 8889S |
| **Cell Lines** |  |  |
| Human iPSC-derive astrocyte progenitors | Axol Bioscience | ax0081 |
| Human iPSC-derive hepatocytes | Takara Bio | Y10133 |
| Human iPSC-derive pancreatic β-cells | Takara Bio | Y10100 |
| A549 Human lung carcinoma cell line | ATCC | CCL-185 |
| U-251 MG Human glioblastoma cell line | Sigma | 9063001 |
| **Human RNA Samples** |  |  |
| Human Brain Total RNA | Takara Bio | 636530 |
| Human Liver Total RNA | Takara Bio | 636531 |
| Human Small Intestine Total RNA | Takara Bio | 636539 |
| Human Colon Total RNA | Takara Bio | 636553 |
| Human Pancreas Total RNA | Takara Bio | 636577 |
| Human Stomach Total RNA | Takara Bio | 636578 |
| Human Brain, Whole Marathon®-Ready cDNA | Takara Bio | 639300 |
| **Oligonucleotides** |  |  |
| qRT-PCR primers for TCF7L2 transcript variants | IDT | see **Table S4** |
| RT-PCR primers for amplification of TCF7L2 cDNA | IDT | see **Table S5** |
| qRT-PCR primers for GAPDH | IDT | Hs.PT.39a.22214836 |
| qRT-PCR primers for VCP | IDT | Hs.PT.58.45655286.g |
| qRT-PCR primers for C1orf43 | IDT | Hs.PT.58.3714142 |
| Negative control B Antisense LNA GapmeR ASO | QIAGEN | 339515 LG00000001-DDA |
| ASO1 | QIAGEN | 339511 LG00226568-DDA |
| ASO2 | QIAGEN | 339511 LG00226571-DDA |
| Non-Targeting Control siRNA Pool #1 | Dharmacon (Horizon) | D-001206-13-05 |
| TCF7L2 (6934) siRNA - SMARTpool | Dharmacon (Horizon) | M-003816-02-0005 |
| **Datasets, Software and Algorithms** |  |  |
| Human brain snRNA-seq dataset | Allen Institute | <https://portal.brain-map.org/atlases-and-data/rnaseq> |
| Human brain scRNA-seq dataset1 | *Darmanis S. et al.* 2017^1^ | GEO accession: GSE84465 |
| Human brain scRNA-seq dataset2 | *Darmanis S, et al.* 2015^2^ | GEO accession: GSE67835 |
| Human brain scATAC-seq dataset | *Corces MR, et al.* 2020^3^ | GEO accession: GSE147672 |
| HaploReg v4.1 | Broad Institute | <https://pubs.broadinstitute.org/mammals/haploreg/haploreg.php> |
| FIMO | The MEME Suite | <https://meme-suite.org/meme/tools/fimo> |
| FastQC | Babraham Institue | <https://www.bioinformatics.babraham.ac.uk/projects/fastqc/> |
| STAR | *Dobin A, et al*. 2013^4^ | <https://github.com/alexdobin/STAR/releases> |
| edgeR | *Robinson MD, et al*. 2010^5^ | <https://bioconductor.org/packages/release/bioc/html/edgeR.html> |
| Enrichr | *Kuleshov MV, et al*. 2016^6^ | <https://maayanlab.cloud/Enrichr/> |
| Gene Ontology | *Ashburner M, et al*. 2000^7^ | <http://geneontology.org/> |
| deepTools2 | *Ramirez F, et al*. 2016^8^ | [https://deeptools.readthedocs.io/en/develop/index.html#](https://deeptools.readthedocs.io/en/develop/index.html) |
| BETA | *Wang S, et al*. 2013^9^ | <http://cistrome.dfci.harvard.edu/BETA/> |

^1-9^See *References* at the end of all Supplementary Tables.

**Table S2**. **Human *TCF7L2* Transcript Variants Annotated by Ensembl**

| Transcript ID | Transcript Name | Length (bp) | Alternative Exon Junction(s)^a^ |
| --- | --- | --- | --- |
| ENST00000277945.11 | TCF7L2-201 | 620 | J11-13 |
| ENST00000346198.5 | TCF7L2-202 | 667 | J3-3a, J3a-4, J4-4a', J4a-5 |
| ENST00000349937.7 | TCF7L2-203 | 725 | **J4b-5** |
| ENST00000352065.10 | TCF7L2-204 | 1553 | J13-13b, J13b-14 |
| ENST00000355717.9 | TCF7L2-205 | 1979 | J4-4a', J4a-5, J11-14 |
| ENST00000355995.8 | TCF7L2-206 | 4073 | J3-3a, J3a-4 |
| ENST00000369386.5 | TCF7L2-207 | 1274 | J13-13a, J13a-14 |
| ENST00000369389.6 | TCF7L2-208 | 691 | **J4d-5** |
| ENST00000369395.6 | TCF7L2-209 | 1541 | J6'-7, J13-13b, J13b-14 |
| ENST00000369397.8 | TCF7L2-210 | 3802 | N/A |
| ENST00000466338.5 | TCF7L2-211 | 1026 | J11-14 |
| ENST00000470254.1 | TCF7L2-212 | 768 | J11-13, J13-13a, **J13a-13b**, J13b-14 |
| ENST00000471569.1 | TCF7L2-213 | 330 | J11-14 |
| ENST00000480888.1 | TCF7L2-214 | 182 | **J11-14'** |
| ENST00000494353.1 | TCF7L2-215 | 500 | J13b-14 |
| ENST00000534894.5 | TCF7L2-216 | 3778 | **J3-5**, J11-14 |
| ENST00000536810.5 | TCF7L2-217 | 3953 | J11-13 |
| ENST00000538897.5 | TCF7L2-218 | 4000 | J3-3a, J3a-4, **J12-14** |
| ENST00000542695.5 | TCF7L2-219 | 4136 | J4-4a, J4a-5, J6'-7, **J12-13a**, J13a-14 |
| ENST00000543371.5 | TCF7L2-220 | 4037 | J3-3a, J3a-4, J7-8', J11-13a, J13a-14 |
| ENST00000545257.6 | TCF7L2-221 | 2160 | J7-8', J13-13a, J13a-14 |
| ENST00000627217.3 | TCF7L2-222 | 4025 | J3-3a, J3a-4, J11-13a, J13a-14 |
| ENST00000629706.2 | TCF7L2-223 | 1755 | J7-8', J11-13a, J13a-14 |
| ENST00000636236.1 | TCF7L2-224 | 582 | **J4f3-5** |
| ENST00000636309.1 | TCF7L2-225 | 563 | **J4g-5** |
| ENST00000636447.1 | TCF7L2-226 | 550 | **J4f1-5** |
| ENST00000636585.1 | TCF7L2-227 | 495 | **J4h-4i, J4i-5** |
| ENST00000637321.1 | TCF7L2-228 | 430 | **J4c-5** |
| ENST00000637416.1 | TCF7L2-229 | 576 | **J4e-5,** J6'-7 |
| ENST00000637574.1 | TCF7L2-230 | 933 | **J4f2-5,** J6'-7 |

^a^ Alternative exon junctions were defined as exon junctions which differed from those of the TCF7L2 reference transcript (ID: ENST00000369397.8). Exon junctions (J) were named based on the exon numbers assigned in **Fig. 2a**. Unique junctions for a specific transcript variant are bold. N/A = not applicable, which means that an alternative junction was not applicable to the reference transcript based on the definition of an alternative junction.

**Table S3. Human TCF7L2 Exons Annotated by the Ensembl**

| Exon ID | Exon Number ^a^ | Exon Position (bp) ^b^ |
| --- | --- | --- |
| ENSE00002258101.1 | Exon1 | chr10:112950250-112950945 |
| ENSE00003644651.1 | Exon2 | chr10:112951207-112951273 |
| ENSE00003496147.1 | Exon3 | chr10:112951483-112951607 |
| ENSE00000987139.1 | Exon3a | chr10:112964556-112964624 |
| ENSE00003734737.1 | Exon4 | chr10:113040025-113040126 |
| ENSE00001449946.1 | Exon4a | chr10:113089397-113089540 |
| ENSE00003792716.1 | Exon4b | chr10:113125536-113125938 |
| ENSE00003798633.1 | Exon4c | chr10:113126022-113126102 |
| ENSE00003795289.1 | Exon4d | chr10:113126690-113126881 |
| ENSE00003793465.1 | Exon4e | chr10:113126957-113127053 |
| ENSE00003792476.1 | Exon4f | chr10:113129206-113129987 |
| ENSE00003792851.1 | Exon4g | chr10:113132022-113132101 |
| ENSE00003799222.1 | Exon4h | chr10:113132896-113133020 |
| ENSE00003793077.1 | Exon4i | chr10:113133240-113133289 |
| ENSE00003789188.1 | Exon5 | chr10:113141184-113141316 |
| ENSE00003644692.1 | Exon6 | chr10:113143923-113144025 |
| ENSE00003527108.1 | Exon7 | chr10:113146011-113146097 |
| ENSE00001699148.1 | Exon8 | chr10:113150983-113151123 |
| ENSE00003517323.1 | Exon9 | chr10:113151725-113151884 |
| ENSE00003524030.1 | Exon10 | chr10:113152333-113152440 |
| ENSE00003620069.1 | Exon11 | chr10:113158021-113158069 |
| ENSE00003590241.1 | Exon12 | chr10:113158667-113158717 |
| ENSE00003601851.1 | Exon13 | chr10:113159920-113159992 |
| ENSE00003487390.1 | Exon13a | chr10:113160619-113160691 |
| ENSE00003603894.1 | Exon13b | chr10:113161579-113161603 |
| ENSE00003461640.1 | Exon14 | chr10:113165555-113167678 |

^a^ Exons were numbered based on the *TCF7L2* reference RNA transcript (ENST00000369397). Alternative exons that map to intronic regions of the reference RNA transcript were named with the intron number followed by a letter of the alphabet (see **Fig. 2a**). ^b^ Numbers are base pair (bp) positions mapped to the human reference genome (GRCh38/p13) for chromosome 10 (chr10).

**Table S4. Human TCF7L2 Exon Junctions Annotated by Ensembl and PCR Primers used to Amplify Specific Junctions**.

| Junction Position (bp)^a^ | Junction Number ^b^ | PCR Primers Used to Amplify Exon Junctions^c^ | | |
| --- | --- | --- | --- | --- |
|  |  | Forward Primer | Reverse Primer | Amplicon Size (bp) |
| chr10:112950946-112951206 | J1-2 | TCAAAACAGCTCCTCCGATT | AAACTTTCCCGGGATTTGTC | 84 |
| chr10:112951274-112951482 | J2-3 | AGACAAATCCCGGGAAAGTT | GGGATCATGATGAAGGGGTA | 99 |
| chr10:112951608-112964555 | J3-3a | GTATCCCGGCTACCCCTTC | GTAATGTGTGCTGCCGGACT | 115 |
| chr10:112951608-113040024 | J3-4 | GTATCCCGGCTACCCCTTC | GTGGCCATTTCATCTGGAGA | 107 |
| chr10:112964625-113040024 | J3a-4 | AGTCCGGCAGCACACATTAC | GTGGCCATTTCATCTGGAGA | 81 |
| chr10:112951608-113141183 | J3-5 | GCCCGAACCTCTAACAAAGT | GCGTGAAGTGTTCATTGCTG | 94 |
| chr10:113040127-113089396 | J4-4a | GGCACACATTGTCCAGAGC | CCCTGAAGACTTGAGTGCTG | 104 |
| chr10:113040127-113089399 | J4-4a' | TCACCGGCACACATTGTCA | CCCTGAAGACTTGAGTGCTG | 105 |
| chr10:113040127-113141183 | J4-5 | CCGGCACACATTGTCTCTAA | GCGTGAAGTGTTCATTGCTG | 100 |
| chr10:113089541-113141183 | J4a-5 | GGACATGAAAAGGAGCCACT | ACGTGATAAGAGGCGTGAGG | 119 |
| chr10:113125939-113141183 | J4b-5 | TGACACCTCTGATGCAGTTTCT | GCACCACTGGCACTTTGTTA | 88 |
| chr10:113126103-113141183 | J4c-5 | CGCAGCGGTGGTTTAATATG | GCACCACTGGCACTTTGTTA | 85 |
| chr10:113126882-113141183 | J4d-5 | CCGCAACCCTCTCTAGATGT | GCGTGAAGTGTTCATTGCTG | 104 |
| chr10:113127054-113141183 | J4e-5 | CCATGTTCGCTGTCACTTTG | ACGTGATAAGAGGCGTGAGG | 115 |
| chr10:113129373-113141183 | J4f1-5 | GATAAAGTGCCTGCCTCCTG | GCACCACTGGCACTTTGTTA | 123 |
| chr10:113129988-113141183 | J4f2-5 | TGGGAATGGAAGATTTGAGTG | GCACCACTGGCACTTTGTTA | 120 |
| chr10:113129815-113141183 | J4f3-5 | AGCAGAAAGGGAGGAGGAAG | ACGTGATAAGAGGCGTGAGG | 111 |
| chr10:113132102-113141183 | J4g-5 | GATGCCCATGTTTGGGATAC | GCACCACTGGCACTTTGTTA | 85 |
| chr10:113133021-113133239 | J4h-4i | AACCCTGGAAACTCGCTGAT | N/D | 124 |
| chr10:113133290-113141183 | J4i-5 | N/D | GCACCACTGGCACTTTGTTA |  |
| chr10:113141317-113143922 | J5-6 | TCACGTACAGCAATGAACACT | CGGGGATATATCTGGAGGGT | 113 |
| chr10:113144014-113146010 | J6'-7 | CATCCGCTAGGATGGCAAG | CTGGACATGGAAGCATTGAC | 101 |
| chr10:113144026-113146010 | J6-7 | GGATGGTTAGTACCACAGCAAG | CTGGACATGGAAGCATTGAC | 104 |
| chr10:113146098-113150982 | J7-8' | GCTTCCATGTCCAGCTTTCT | CACTCTGGGACGATTCCTGT | 133 |
| chr10:113146098-113150997 | J7-8 | TGTACCCAATCACGACAGGA | CATATGGGGAGGGAACCTG | 89 |
| chr10:113151124-113151724 | J8-9 | CATATGGTCCCACCACATCA | TGGAGTCCTGATGCTTTGAAC | 133 |
| chr10:113151885-113152332 | J9-10 | N/D | N/D |  |
| chr10:113152441-113158020 | J10-11 | N/D | N/D |  |
| chr10:113158070-113158666 | J11-12 | AAGAGGAAAAGGGACAAGCA | TGTAATCGGAGGAAGTGAAAGG | 87 |
| chr10:113158070-113159919 | J11-13 | CGGGAGAGACCAATGACCT | GCCGCACCAGTTATTCTGTT | 80 |
| chr10:113158070-113160618 | J11-13a | AAGAGGAAAAGGGACAAGCAG | CACGGTTTGCACCATAAAGTC | 107 |
| chr10:113158070-113165554 | J11-14 | GGGAGAGACCAATGGAGAA | ATCTGAAGAGGGTGGGCTGA | 81 |
| chr10:113158070-113165855 | J11-14' | AAGAGGAAAAGGGACAAGCA | GGAGCTGTGGGAATGTAAGG | 100 |
| chr10:113158718-113159919 | J12-13 | TCCTTGCCTTTCACTTCCTC | GCCGCACCAGTTATTCTGTT | 94 |
| chr10:113158718-113160618 | J12-13a | TCCTTGCCTTTCACTTCCTC | CACGGTTTGCACCATAAAGTC | 99 |
| chr10:113158718-113165554 | J12-14 | TCCTCCGATTACAGGAGAAAAA | GGGGGAGGCGAATCTAGTAA | 107 |
| chr10:113159993-113160618 | J13-13a | TGAGCGCTCCTAAGAAATGC | ACAGTGCCCGACACTTCTTT | 103 |
| chr10:113159993-113161578 | J13-13b | TGAGCGCTCCTAAGAAATGC | CCAAATTCAAAGACTGCAAGG | 83 |
| chr10:113159993-113165554 | J13-14 | TGAGCGCTCCTAAGAAATGC | GCAGCTGCCTTCACCTTGTA | 116 |
| chr10:113160692-113161578 | J13a-13b | GCAAATACTCCAAAGAAGTGTCG | TTCCAAATTCAAAGACTGCAC | 86 |
| chr10:113160692-113165554 | J13a-14 | N/D | N/D |  |
| chr10:113161604-113165554 | J13b-14 | CCACCTTTGGTTAAATGTGTTG | GCAGCTGCCTTCACCTTGTA | 116 |

^a^ Numbers are base pair (bp) positions mapped to the human reference genome (GRCh38/p13) for chromosome 10 (chr10). ^b^ Exon junctions were numbered based on the exon numbers assigned in supplementary **Table S3**. ^c^ Primers used to quantify RNA levels of TCF7L2 transcript variants using qRT-PCR. N/D = not determined because the corresponding junction was not needed to quantify for the identification of TCF7L2 transcript variants when other junctions were determined.

**Table S5. Primers Used in the RT-PCR Assays to Amplify TCF7L2 cDNA.**

| Primer Name^a^ | Targeting Exon(s) | Sequence |
| --- | --- | --- |
| P1 | Exon 1 | TCAAAACAGCTCCTCCGATT |
| P2 | Exon 3 | GTATCCCGGCTACCCCTTC |
| P3 | Exons 4d-5 | CCGCAACCCTCTCTAGATGT |
| P4 | Exon 5 | GCGTGAAGTGTTCATTGCTG |
| P5 | Exon 6 | CGGGGATATATCTGGAGGGT |
| P6 | Exon 7 | CTGGACATGGAAGCATTGAC |
| P7 | Exons 9-8 | TGGAGTCCTGATGCTTTGAAC |
| P8 | Exon 13 | GCCGCACCAGTTATTCTGTT |
| P9 | Exon 14 | GCAGCTGCCTTCACCTTGTA |

^a^ Primer numbers correspond with those labeled in **Fig 3a**.

**Table S6. Differentially-Expressed Genes after "T-3" KD in hiPSC-derived Astrocytes.** (see attached Excel file)

**Table S7. TCF7L2 Target Genes Identified by Integration of RNA-seq and ChIP-seq Data.** (see attached Excel file)

**Table S8. TCF7L2 Regulated Bipolar Disorder (BD) Risk Genes in Human iPSC-derived Astrocytes: Function and Genetically-Associated Phenotypes.**

| Gene Name | Relation to BD | Gene Function | Other Phenotype(s) in GWAS^a^ |
| --- | --- | --- | --- |
| *NCAN* | - Associated with BD in GWAS ^10, 11^; - Differentially expressed in iPSC-derived cerebral organoids from BD patients ^12^. | Encodes neurocan, a chondroitin sulfate proteoglycan of brain ^13^; Modulate neuronal adhesion and neurite growth during development ^14^. | Plasma low density lipoprotein (LDL) cholesterol and triglycerides levels ^15, 16^. |
| *TENM4 (ODZ4)* | Associated with BD in GWAS. ^17-20^**.** | Encodes teneurin-4 which is a novel regulator of oligodendrocyte differentiation and myelination of small-diameter axons in the CNS ^21^. | - Schizophrenia (SCZ) ^22^; - Self-reported educational attainment, cognitive performance ^23^; - Intelligence ^24^. |
| *ZMIZ1* | - Associated with BD in GWAS ^17, 25, 26^; - Expression level in human brain associated with BD ^27^ | Acts as transcriptional coactivator. Involved in regulation of postmitotic positioning of pyramidal neurons in the developing cerebral cortex. ZMIZ1 variants cause a syndromic neurodevelopmental disorder. ^28^ | - Type 2 diabetes ^29-35^; - Body mass index (BMI)-adjusted waist circumference ^36, 37^; - BMI-adjusted waist-hip ratio ^24^; - Body height ^38^; - Triglyceride levels ^39^. |
| *ZSWIM6* | Associated with BD in GWAS ^40^ | Involved in nervous system development, important for striatal morphology and motor regulation ^41^; Mutation results in severe intellectual disability ^42^. | - BMI ^37, 38, 43^; - SCZ ^22, 44^; - Self-reported educational attainment and general cognitive ability ^23, 45-47^; - Brain region volumes ^48^. |
| *DOK5* | Associated with amygdala activation in youths with bipolar disorder in GWAS ^49^. | Encodes insulin receptor substrate 6 ^50^; Mediates neuronal differentiation through c-Ret receptor tyrosine kinase ^51^. | BMI ^37, 38^ |
| *FBLN1* | Associated with temperament in BD in GWAS ^52^. | Extracellular glycoprotein that plays a role in cell adhesion and motility along fibers within the ECM ^53^; Required for morphogenesis of neural crest-derived structure ^54^. | LDL cholesterol level ^55^. |
| *NFIA* | Associated with BD in GWAS^56, 57^ | NFIA is a gliogenic switch enabling rapid derivation of human astrocytes from pluripotent stem cells ^58^; Transcriptional regulation during the initiation of gliogenesis^59^ | - Neuroticism ^60^; - Self-reported educational attainment, cognitive performance ^23^; - High density lipoprotein cholesterol and triglyceride levels ^15, 39^; - Body height ^38^. |
| *SLC45A4* | - Associated with BD in GWAS ^20^; - Risk genes for BD in young adult ^61^. | Sucrose transporter ^62^ | - Body height ^38^; - Birth weight ^63, 64^ |

^a^Data obtained from the GWAS catalog. All phenotypes listed in the table were associated with SNPs at genome-wide significant levels (p-value < 5E-08).

**References**:

1. Darmanis S, Sloan SA, Croote D, Mignardi M, Chernikova S, Samghababi P *et al.* Single-Cell RNA-Seq Analysis of Infiltrating Neoplastic Cells at the Migrating Front of Human Glioblastoma. *Cell Rep* 2017; **21**(5)**:** 1399-1410.

2. Darmanis S, Sloan SA, Zhang Y, Enge M, Caneda C, Shuer LM *et al.* A survey of human brain transcriptome diversity at the single cell level. *Proc Natl Acad Sci U S A* 2015; **112**(23)**:** 7285-7290.

3. Corces MR, Shcherbina A, Kundu S, Gloudemans MJ, Fresard L, Granja JM *et al.* Single-cell epigenomic analyses implicate candidate causal variants at inherited risk loci for Alzheimer's and Parkinson's diseases. *Nat Genet* 2020; **52**(11)**:** 1158-1168.

4. Dobin A, Davis CA, Schlesinger F, Drenkow J, Zaleski C, Jha S *et al.* STAR: ultrafast universal RNA-seq aligner. *Bioinformatics* 2013; **29**(1)**:** 15-21.

5. Robinson MD, McCarthy DJ, Smyth GK. edgeR: a Bioconductor package for differential expression analysis of digital gene expression data. *Bioinformatics* 2010; **26**(1)**:** 139-140.

6. Kuleshov MV, Jones MR, Rouillard AD, Fernandez NF, Duan Q, Wang Z *et al.* Enrichr: a comprehensive gene set enrichment analysis web server 2016 update. *Nucleic Acids Res* 2016; **44**(W1)**:** W90-97.

7. Ashburner M, Ball CA, Blake JA, Botstein D, Butler H, Cherry JM *et al.* Gene ontology: tool for the unification of biology. The Gene Ontology Consortium. *Nat Genet* 2000; **25**(1)**:** 25-29.

8. Ramirez F, Ryan DP, Gruning B, Bhardwaj V, Kilpert F, Richter AS *et al.* deepTools2: a next generation web server for deep-sequencing data analysis. *Nucleic Acids Res* 2016; **44**(W1)**:** W160-165.

9. Wang S, Sun H, Ma J, Zang C, Wang C, Wang J *et al.* Target analysis by integration of transcriptome and ChIP-seq data with BETA. *Nat Protoc* 2013; **8**(12)**:** 2502-2515.

10. Stahl EA, Breen G, Forstner AJ, McQuillin A, Ripke S, Trubetskoy V *et al.* Genome-wide association study identifies 30 loci associated with bipolar disorder. *Nat Genet* 2019; **51**(5)**:** 793-803.

11. Cichon S, Muhleisen TW, Degenhardt FA, Mattheisen M, Miro X, Strohmaier J *et al.* Genome-wide association study identifies genetic variation in neurocan as a susceptibility factor for bipolar disorder. *Am J Hum Genet* 2011; **88**(3)**:** 372-381.

12. Kathuria A, Lopez-Lengowski K, Vater M, McPhie D, Cohen BM, Karmacharya R. Transcriptome analysis and functional characterization of cerebral organoids in bipolar disorder. *Genome Med* 2020; **12**(1)**:** 34.

13. Rauch U, Feng K, Zhou XH. Neurocan: a brain chondroitin sulfate proteoglycan. *Cell Mol Life Sci* 2001; **58**(12-13)**:** 1842-1856.

14. Friedlander DR, Milev P, Karthikeyan L, Margolis RK, Margolis RU, Grumet M. The neuronal chondroitin sulfate proteoglycan neurocan binds to the neural cell adhesion molecules Ng-CAM/L1/NILE and N-CAM, and inhibits neuronal adhesion and neurite outgrowth. *J Cell Biol* 1994; **125**(3)**:** 669-680.

15. Hoffmann TJ, Theusch E, Haldar T, Ranatunga DK, Jorgenson E, Medina MW *et al.* A large electronic-health-record-based genome-wide study of serum lipids. *Nat Genet* 2018; **50**(3)**:** 401-413.

16. Wojcik GL, Graff M, Nishimura KK, Tao R, Haessler J, Gignoux CR *et al.* Genetic analyses of diverse populations improves discovery for complex traits. *Nature* 2019; **570**(7762)**:** 514-518.

17. Cross-Disorder Group of the Psychiatric Genomics C. Identification of risk loci with shared effects on five major psychiatric disorders: a genome-wide analysis. *Lancet* 2013; **381**(9875)**:** 1371-1379.

18. Ikeda M, Takahashi A, Kamatani Y, Okahisa Y, Kunugi H, Mori N *et al.* A genome-wide association study identifies two novel susceptibility loci and trans population polygenicity associated with bipolar disorder. *Mol Psychiatry* 2018; **23**(3)**:** 639-647.

19. Muhleisen TW, Leber M, Schulze TG, Strohmaier J, Degenhardt F, Treutlein J *et al.* Genome-wide association study reveals two new risk loci for bipolar disorder. *Nat Commun* 2014; **5:** 3339.

20. PsychiatricGWASConsortiumBipolarDisorderWorkingGroup. Large-scale genome-wide association analysis of bipolar disorder identifies a new susceptibility locus near ODZ4. *Nat Genet* 2011; **43**(10)**:** 977-983.

21. Suzuki N, Fukushi M, Kosaki K, Doyle AD, de Vega S, Yoshizaki K *et al.* Teneurin-4 is a novel regulator of oligodendrocyte differentiation and myelination of small-diameter axons in the CNS. *J Neurosci* 2012; **32**(34)**:** 11586-11599.

22. Lam M, Chen CY, Li Z, Martin AR, Bryois J, Ma X *et al.* Comparative genetic architectures of schizophrenia in East Asian and European populations. *Nat Genet* 2019; **51**(12)**:** 1670-1678.

23. Lee JJ, Wedow R, Okbay A, Kong E, Maghzian O, Zacher M *et al.* Gene discovery and polygenic prediction from a genome-wide association study of educational attainment in 1.1 million individuals. *Nat Genet* 2018; **50**(8)**:** 1112-1121.

24. Lotta LA, Wittemans LBL, Zuber V, Stewart ID, Sharp SJ, Luan J *et al.* Association of Genetic Variants Related to Gluteofemoral vs Abdominal Fat Distribution With Type 2 Diabetes, Coronary Disease, and Cardiovascular Risk Factors. *JAMA* 2018; **320**(24)**:** 2553-2563.

25. Liu Y, Blackwood DH, Caesar S, de Geus EJ, Farmer A, Ferreira MA *et al.* Meta-analysis of genome-wide association data of bipolar disorder and major depressive disorder. *Mol Psychiatry* 2011; **16**(1)**:** 2-4.

26. Ferreira MA, O'Donovan MC, Meng YA, Jones IR, Ruderfer DM, Jones L *et al.* Collaborative genome-wide association analysis supports a role for ANK3 and CACNA1C in bipolar disorder. *Nat Genet* 2008; **40**(9)**:** 1056-1058.

27. McCarthy MJ, Liang S, Spadoni AD, Kelsoe JR, Simmons AN. Whole brain expression of bipolar disorder associated genes: structural and genetic analyses. *PLoS One* 2014; **9**(6)**:** e100204.

28. Carapito R, Ivanova EL, Morlon A, Meng L, Molitor A, Erdmann E *et al.* ZMIZ1 Variants Cause a Syndromic Neurodevelopmental Disorder. *Am J Hum Genet* 2019; **104**(2)**:** 319-330.

29. Morris AP, Voight BF, Teslovich TM, Ferreira T, Segre AV, Steinthorsdottir V *et al.* Large-scale association analysis provides insights into the genetic architecture and pathophysiology of type 2 diabetes. *Nat Genet* 2012; **44**(9)**:** 981-990.

30. Replication DIG, Meta-analysis C, Asian Genetic Epidemiology Network Type 2 Diabetes C, South Asian Type 2 Diabetes C, Mexican American Type 2 Diabetes C, Type 2 Diabetes Genetic Exploration by Nex-generation sequencing in muylti-Ethnic Samples C *et al.* Genome-wide trans-ancestry meta-analysis provides insight into the genetic architecture of type 2 diabetes susceptibility. *Nat Genet* 2014; **46**(3)**:** 234-244.

31. Zhao W, Rasheed A, Tikkanen E, Lee JJ, Butterworth AS, Howson JMM *et al.* Identification of new susceptibility loci for type 2 diabetes and shared etiological pathways with coronary heart disease. *Nat Genet* 2017; **49**(10)**:** 1450-1457.

32. Mahajan A, Taliun D, Thurner M, Robertson NR, Torres JM, Rayner NW *et al.* Fine-mapping type 2 diabetes loci to single-variant resolution using high-density imputation and islet-specific epigenome maps. *Nat Genet* 2018; **50**(11)**:** 1505-1513.

33. Xue A, Wu Y, Zhu Z, Zhang F, Kemper KE, Zheng Z *et al.* Genome-wide association analyses identify 143 risk variants and putative regulatory mechanisms for type 2 diabetes. *Nat Commun* 2018; **9**(1)**:** 2941.

34. Flannick J, Mercader JM, Fuchsberger C, Udler MS, Mahajan A, Wessel J *et al.* Exome sequencing of 20,791 cases of type 2 diabetes and 24,440 controls. *Nature* 2019; **570**(7759)**:** 71-76.

35. Vujkovic M, Keaton JM, Lynch JA, Miller DR, Zhou J, Tcheandjieu C *et al.* Discovery of 318 new risk loci for type 2 diabetes and related vascular outcomes among 1.4 million participants in a multi-ancestry meta-analysis. *Nat Genet* 2020; **52**(7)**:** 680-691.

36. Graff M, Scott RA, Justice AE, Young KL, Feitosa MF, Barata L *et al.* Genome-wide physical activity interactions in adiposity - A meta-analysis of 200,452 adults. *PLoS Genet* 2017; **13**(4)**:** e1006528.

37. Pulit SL, Stoneman C, Morris AP, Wood AR, Glastonbury CA, Tyrrell J *et al.* Meta-analysis of genome-wide association studies for body fat distribution in 694 649 individuals of European ancestry. *Hum Mol Genet* 2019; **28**(1)**:** 166-174.

38. Kichaev G, Bhatia G, Loh PR, Gazal S, Burch K, Freund MK *et al.* Leveraging Polygenic Functional Enrichment to Improve GWAS Power. *Am J Hum Genet* 2019; **104**(1)**:** 65-75.

39. Richardson TG, Sanderson E, Palmer TM, Ala-Korpela M, Ference BA, Davey Smith G *et al.* Evaluating the relationship between circulating lipoprotein lipids and apolipoproteins with risk of coronary heart disease: A multivariable Mendelian randomisation analysis. *PLoS Med* 2020; **17**(3)**:** e1003062.

40. Wu Y, Cao H, Baranova A, Huang H, Li S, Cai L *et al.* Multi-trait analysis for genome-wide association study of five psychiatric disorders. *Transl Psychiatry* 2020; **10**(1)**:** 209.

41. Tischfield DJ, Saraswat DK, Furash A, Fowler SC, Fuccillo MV, Anderson SA. Loss of the neurodevelopmental gene Zswim6 alters striatal morphology and motor regulation. *Neurobiol Dis* 2017; **103:** 174-183.

42. Palmer EE, Kumar R, Gordon CT, Shaw M, Hubert L, Carroll R *et al.* A Recurrent De Novo Nonsense Variant in ZSWIM6 Results in Severe Intellectual Disability without Frontonasal or Limb Malformations. *Am J Hum Genet* 2017; **101**(6)**:** 995-1005.

43. Akiyama M, Okada Y, Kanai M, Takahashi A, Momozawa Y, Ikeda M *et al.* Genome-wide association study identifies 112 new loci for body mass index in the Japanese population. *Nat Genet* 2017; **49**(10)**:** 1458-1467.

44. Pardinas AF, Holmans P, Pocklington AJ, Escott-Price V, Ripke S, Carrera N *et al.* Common schizophrenia alleles are enriched in mutation-intolerant genes and in regions under strong background selection. *Nat Genet* 2018; **50**(3)**:** 381-389.

45. Okbay A, Beauchamp JP, Fontana MA, Lee JJ, Pers TH, Rietveld CA *et al.* Genome-wide association study identifies 74 loci associated with educational attainment. *Nature* 2016; **533**(7604)**:** 539-542.

46. Davies G, Lam M, Harris SE, Trampush JW, Luciano M, Hill WD *et al.* Study of 300,486 individuals identifies 148 independent genetic loci influencing general cognitive function. *Nat Commun* 2018; **9**(1)**:** 2098.

47. Hill WD, Marioni RE, Maghzian O, Ritchie SJ, Hagenaars SP, McIntosh AM *et al.* A combined analysis of genetically correlated traits identifies 187 loci and a role for neurogenesis and myelination in intelligence. *Mol Psychiatry* 2019; **24**(2)**:** 169-181.

48. Zhao B, Luo T, Li T, Li Y, Zhang J, Shan Y *et al.* Genome-wide association analysis of 19,629 individuals identifies variants influencing regional brain volumes and refines their genetic co-architecture with cognitive and mental health traits. *Nat Genet* 2019; **51**(11)**:** 1637-1644.

49. Liu X, Akula N, Skup M, Brotman MA, Leibenluft E, McMahon FJ. A genome-wide association study of amygdala activation in youths with and without bipolar disorder. *J Am Acad Child Adolesc Psychiatry* 2010; **49**(1)**:** 33-41.

50. Cai D, Dhe-Paganon S, Melendez PA, Lee J, Shoelson SE. Two new substrates in insulin signaling, IRS5/DOK4 and IRS6/DOK5. *J Biol Chem* 2003; **278**(28)**:** 25323-25330.

51. Grimm J, Sachs M, Britsch S, Di Cesare S, Schwarz-Romond T, Alitalo K *et al.* Novel p62dok family members, dok-4 and dok-5, are substrates of the c-Ret receptor tyrosine kinase and mediate neuronal differentiation. *J Cell Biol* 2001; **154**(2)**:** 345-354.

52. Greenwood TA, Akiskal HS, Akiskal KK, Bipolar Genome S, Kelsoe JR. Genome-wide association study of temperament in bipolar disorder reveals significant associations with three novel Loci. *Biol Psychiatry* 2012; **72**(4)**:** 303-310.

53. Twal WO, Czirok A, Hegedus B, Knaak C, Chintalapudi MR, Okagawa H *et al.* Fibulin-1 suppression of fibronectin-regulated cell adhesion and motility. *J Cell Sci* 2001; **114**(Pt 24)**:** 4587-4598.

54. Cooley MA, Kern CB, Fresco VM, Wessels A, Thompson RP, McQuinn TC *et al.* Fibulin-1 is required for morphogenesis of neural crest-derived structures. *Dev Biol* 2008; **319**(2)**:** 336-345.

55. Klimentidis YC, Arora A, Newell M, Zhou J, Ordovas JM, Renquist BJ *et al.* Phenotypic and Genetic Characterization of Lower LDL Cholesterol and Increased Type 2 Diabetes Risk in the UK Biobank. *Diabetes* 2020; **69**(10)**:** 2194-2205.

56. Lee HJ, Woo HG, Greenwood TA, Kripke DF, Kelsoe JR. A genome-wide association study of seasonal pattern mania identifies NF1A as a possible susceptibility gene for bipolar disorder. *J Affect Disord* 2013; **145**(2)**:** 200-207.

57. Gonzalez S, Gupta J, Villa E, Mallawaarachchi I, Rodriguez M, Ramirez M *et al.* Replication of genome-wide association study (GWAS) susceptibility loci in a Latino bipolar disorder cohort. *Bipolar Disord* 2016; **18**(6)**:** 520-527.

58. Tchieu J, Calder EL, Guttikonda SR, Gutzwiller EM, Aromolaran KA, Steinbeck JA *et al.* NFIA is a gliogenic switch enabling rapid derivation of functional human astrocytes from pluripotent stem cells. *Nat Biotechnol* 2019; **37**(3)**:** 267-275.

59. Kang P, Lee HK, Glasgow SM, Finley M, Donti T, Gaber ZB *et al.* Sox9 and NFIA coordinate a transcriptional regulatory cascade during the initiation of gliogenesis. *Neuron* 2012; **74**(1)**:** 79-94.

60. Nagel M, Jansen PR, Stringer S, Watanabe K, de Leeuw CA, Bryois J *et al.* Meta-analysis of genome-wide association studies for neuroticism in 449,484 individuals identifies novel genetic loci and pathways. *Nat Genet* 2018; **50**(7)**:** 920-927.

61. Fries GR, Quevedo J, Zeni CP, Kazimi IF, Zunta-Soares G, Spiker DE *et al.* Integrated transcriptome and methylome analysis in youth at high risk for bipolar disorder: a preliminary analysis. *Transl Psychiatry* 2017; **7**(3)**:** e1059.

62. Bartolke R, Heinisch JJ, Wieczorek H, Vitavska O. Proton-associated sucrose transport of mammalian solute carrier family 45: an analysis in Saccharomyces cerevisiae. *Biochem J* 2014; **464**(2)**:** 193-201.

63. Horikoshi M, Beaumont RN, Day FR, Warrington NM, Kooijman MN, Fernandez-Tajes J *et al.* Genome-wide associations for birth weight and correlations with adult disease. *Nature* 2016; **538**(7624)**:** 248-252.

64. Warrington NM, Beaumont RN, Horikoshi M, Day FR, Helgeland O, Laurin C *et al.* Maternal and fetal genetic effects on birth weight and their relevance to cardio-metabolic risk factors. *Nat Genet* 2019; **51**(5)**:** 804-814.
